# The Hippo Pathway Regulates Caveolae Expression and Mediates Flow Response via Caveolae

**DOI:** 10.1016/j.cub.2018.11.066

**Published:** 2019-01-21

**Authors:** Valentina Rausch, Jonathan R. Bostrom, Jiwon Park, Isabel R. Bravo, Yi Feng, David C. Hay, Brian A. Link, Carsten G. Hansen

**Affiliations:** 1 University of Edinburgh Centre for Inflammation Research, Queen’s Medical Research Institute, Edinburgh bioQuarter, 47 Little France Crescent, Edinburgh EH16 4TJ, UK; 2 Department of Cell Biology, Neurobiology and Anatomy, Medical College of Wisconsin, Milwaukee, WI, USA; 3 MRC Centre for Regenerative Medicine, Institute for Regeneration and Repair, University of Edinburgh, Edinburgh bioQuarter, 5 Little France Drive, Edinburgh EH16 4UU, UK

**Keywords:** Hippo pathway, YAP, TAZ, caveolae, CAV1, CAVIN1, mechanotransduction, shear stress, extra cellular matrix

## Abstract

The Hippo pathway plays major roles in development, regeneration, and cancer. Its activity is tightly regulated by both diffusible chemical ligands and mechanical stimuli. The pathway consists of a series of kinases that can control the sub-cellular localization and stability of YAP or TAZ, homologous transcriptional co-factors. Caveolae, small (60–100 nm) bulb-like invaginations of the plasma membrane, are comprised predominantly of caveolin and cavin proteins and can respond to mechanical stimuli. Here, we show that YAP/TAZ, the major transcriptional mediators of the Hippo pathway, are critical for expression of caveolae components and therefore caveolae formation in both mammalian cells and zebrafish. In essence, without YAP/TAZ, the cell loses an entire organelle. *CAVEOLIN1* and *CAVIN1*, the two essential caveolar genes, are direct target genes of YAP/TAZ, regulated via TEA domain (TEAD) transcription factors. Notably, YAP/TAZ become nuclear enriched and facilitate target gene transcription in cells with diminished levels of caveolae. Furthermore, caveolar-mediated shear stress response activates YAP/TAZ. These data link caveolae to Hippo signaling in the context of cellular responses to mechanical stimuli and suggest activity-based feedback regulation between components of caveolae and the outputs of the Hippo pathway.

## Highlights

• YAP/TAZ are critical for CAVIN1 and CAVEOLIN1 expression and caveolae formation
• The essential caveolar genes *CAVIN1* and *CAVEOLIN1* are direct YAP/TAZ-TEAD target genes
• YAP/TAZ are hyperactivated in caveolae-deficient cells
• Caveolae facilitate YAP/TAZ-mediated shear stress response

## Graphical Abstract

**Figure:**
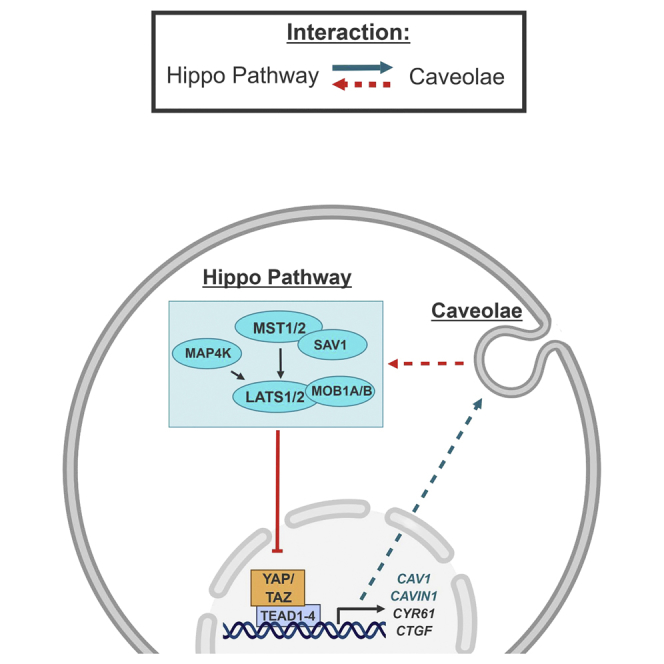

## Teaser

Rausch et al. identifies that the Hippo pathway regulates caveolae abundance via YAP/TAZ-TEAD-mediated transcriptional regulation of the two essential caveolar genes *CAVEOLIN1* and *CAVIN1*. In essence, without YAP/TAZ, the cell loses an entire cellular organelle. In addition, caveolae facilitate shear-stress-mediated signaling via the Hippo pathway.

## Introduction

The Hippo pathway is involved in multiple developmental and regenerative processes, such as tissue renewal of the liver, heart, kidney, intestine, and lung. The Hippo pathway needs to be tightly and dynamically regulated, as otherwise hyperactive yes-associated protein/transcriptional co-activator with PDZ-binding motif (YAP/TAZ) cause disease, most notably cancer [1, 2, 3, 4, 5]. The pathway comprises an upstream inhibitory serine-threonine kinase cascade that ultimately activates large tumor suppressor kinase (LATS)1/2, which in turn phosphorylates and thereby inhibits the co-transcriptional regulators YAP/TAZ through cytoplasmic sequestration as well as protein degradation [6, 7]. The pathway is regulated by both soluble ligands and mechanical stimuli, where the cytoskeleton and Rho-guanosine triphosphatases (GTPases) play central roles in mediating these responses [7, 8]. Importantly, how mechanical signals are sensed at the plasma membrane and transduced via the Hippo pathway is currently not well understood.

Caveolae, meaning little caves, are multifunctional organelles localized in the plasma membrane and have mechanotransductive and protective roles [9, 10, 11, 12, 13, 14, 15]. They are generated by CAVEOLIN1–3 (CAV1–3) and CAVIN1–4 (also known as PTRF, SDPR, PRKCDBP, and MURC) as well as Eps15 homology domain (EHD) and PACSIN2 proteins [16, 17]. CAV3 and CAVIN4 are muscle-specific caveolar proteins [16, 17]. CAV1 is essential for caveolae formation in non-muscle cells, whereas CAVIN1 is critical for caveolae formation in all cells [18, 19, 20, 21].

As both the Hippo pathway and caveolae are regulated by mechanical stimuli, such as shear stress, we sought to investigate whether the Hippo pathway and caveolae are functionally integrated in this process.

## Results

### YAP/TAZ Drive Caveolar Protein Expression

**Figure 1:**
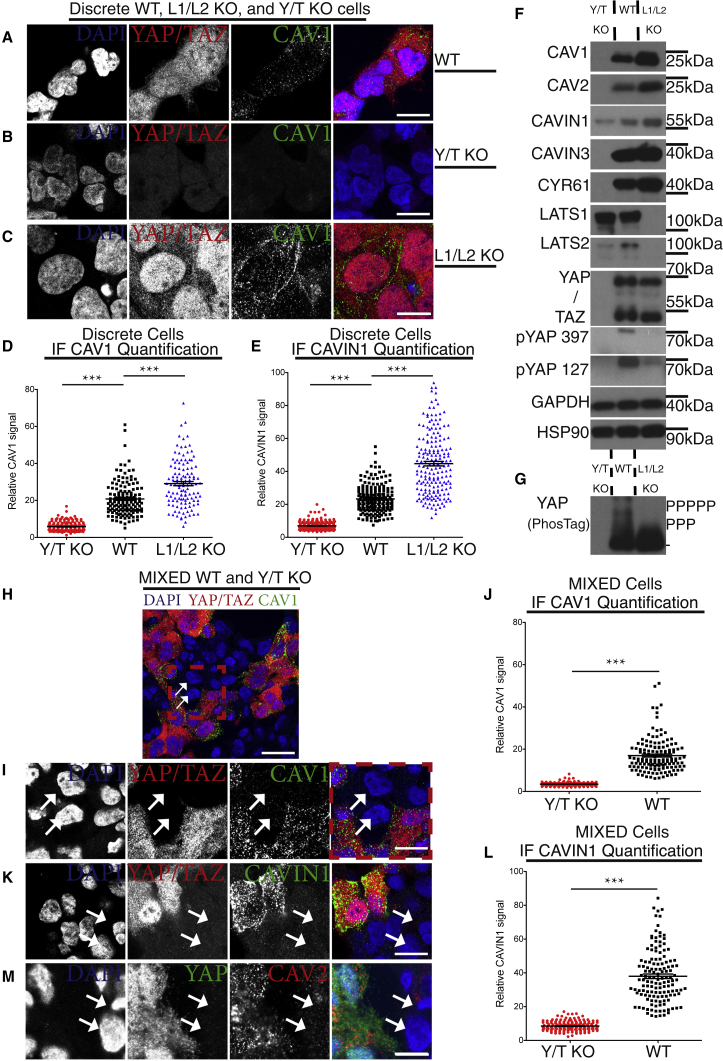
YAP/TAZ Are Necessary for Caveolar Protein Expression (A) Confocal images of wild-type (WT) HEK293A cells labeled for DAPI (blue), YAP/TAZ (red), and CAVEOLIN1 (CAV1) (green). (B and C) YAP/TAZ KO cells (Y/T KO) (B) and LATS1/2 KO (L1/L2 KO) (C) labeled and imaged as cells in (A). Scale bars (A–C) represent 30 μm. (A)–(C) are related to Figures S1A–S1C, S1F, and S2A–S2D. (D) Dot plot of quantified CAV1 levels from images, as shown in (A)–(C). In Y/T KO (red), WT (black), and L1/L2 KO (blue) cells, each dot represents one cell. Means ± SEM. (E) Dot plot of CAVIN1 levels from images as shown in Figure S1B. Means ± SEM. (F) Western blots from Y/T KO, WT, and L1/L2 KO HEK293A cells (Figures S2E and S2G). GAPDH and HSP90 serve as loading controls. (G) PhosTag gel-based western blots probed against YAP from cell lysates as in (F) (Figure S2H). (H) Mixed cell culture of Y/T KO and WT HEK293A cells were fixed and labeled for YAP/TAZ (red), CAV1 (green), and DAPI (blue). Arrows: examples of Y/T KO cells. Note, cells with no YAP/TAZ signal have low CAV1 signal. Scale bar represents 30 μm. (I) Close up of cells from red box in (H). (J) Dot plot of CAV1 levels in mixed cell populations of Y/T KO and WT cells analyzed in images as shown in (H). Each dot represents one cell. Means ± SEM. (K) Mixed cell population as in (H) labeled for YAP/TAZ (red), CAVIN1 (green), and DAPI (blue). Arrows: examples of Y/T KO cells. Zoomed-out image is in Figure S1D. (L) Dot plot of CAVIN1 levels in mixed populations of Y/T KO and WT cells carried out on images as shown in (K). Means ± SEM. (M) Cells as in (H), labeled for YAP (green), CAVEOLIN2 (CAV2) (red), and DAPI (blue). Zoomed-out image is in Figure S1E. Arrows: examples of Y/T KO cells. Scale bars in (I), (K), and (M) are 15 μm. Further related is Figures S7N–S7R.

We utilized genome-edited YAP/TAZ double knockout (Y/T KO) and LATS1/2 double knockout (L1/L2 KO) HEK293A cells [22] and compared these to wild-type (WT) HEK293A cells to examine whether the activity state of YAP/TAZ regulates caveolar protein expression. There was a dramatic reduction in CAVEOLIN and CAVIN protein expression in the Y/T KO cells (Figures 1A, 1B, 1D–1F, and S1A–S1C). This effect was mirrored in an additional Y/T KO clone (Figures S2A, S2B, and S2E). Moreover, caveolar proteins were markedly increased in L1/L2 KO cells (Figures 1A and 1C–1F), where YAP/TAZ are hyperactive, as evident from increased YAP/TAZ nuclear localization (Figures 1A, 1C, S1B, and S1F) and dephosphorylated YAP status (Figures 1F, 1G, and S2E). Re-introduction of LATS1 into L1/L2 KO cells, but not a kinase dead version, increased YAP phosphorylation and decreased CAVIN1, CAV1, and CYR61 expression (Figures S2G and S2H). The essential proteins for caveolae biogenesis, CAV1 and CAVIN1, therefore mirrored the expression of the well-characterized YAP/TAZ target gene *CYR61* [7] (Figures 1F, S2E, and S2G). To determine whether the ability for caveolar protein expression was due to a cell-intrinsic dependence on YAP/TAZ and not merely mediated by paracrine effects, we utilized a mixed cell population immunofluorescence (IF)-based assay. Due to the specificity of the antibodies used (Figure S1A), the assay allowed for direct comparison between Y/T KO cells and WT cells in terms of the CAV1 or CAVIN1 protein levels (Figures 1H–1L, S1D, S1E, S2C, and S2D). CAV1 and CAVIN1 protein expression, as well as CAV2 (Figure 1M), was directly dependent on YAP/TAZ cell-intrinsic expression (Figures 1H–1L). Importantly, upon exogenous plasmid-based re-expression in Y/T KO cells, CAV1 and CAVIN1 could be found co-localizing within plasma membrane domains (Figure S3A). This localization is comparable to that of endogenous CAV1 and CAVIN1 in WT cells (Figure S3B). These data show that YAP/TAZ are required for the expression of the essential caveolar proteins CAVIN1 and CAV1.

### *CAV1* and *CAVIN1* Are Direct YAP/TAZ-TEAD Target Genes

**Figure 2:**
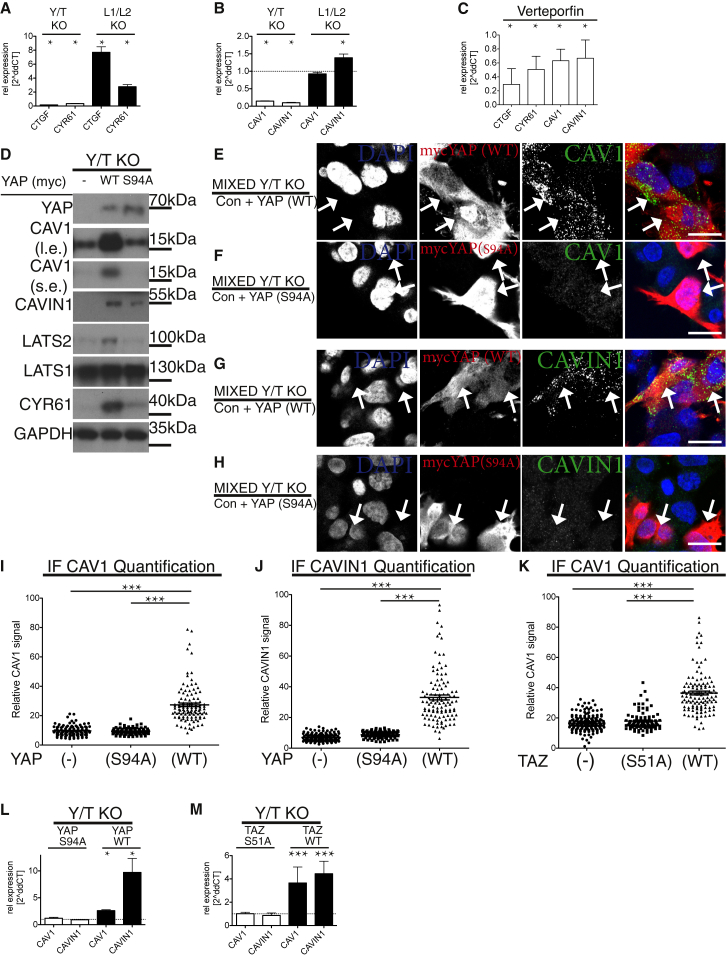
*CAV1* and *CAVIN1* Expression Depend on YAP/TAZ-TEAD Interaction (A) qPCR analysis of established YAP/TAZ targets. mRNA from Y/T KO and L1/L2 KO HEK293A cells compared to WT HEK293A cells (Figures S2F and S2I). Means ± SD. (B) qPCR data as in (A), analyzed for the expression of *CAV1* and *CAVIN1* (Figures S2F and S2I). Means ± SD. (C) Verteporfin-treated WT HEK293A cells analyzed by qPCR. Inhibition of the interaction between YAP-TEAD reduces expression of caveolar genes. Means ± SD. (D) Western blots of lysates from Y/T KO cells stably re-expressing either control plasmid, myc-tagged WT, or S94A mutant YAP. l.e., long exposure; s.e., short exposure. Note, re-expression of WT YAP, but not S94A mutant, deficient in TEAD binding, restores expression of caveolar as well as established YAP/TAZ-TEAD target genes, *CYR61* and *LATS2*. (E and F) Mixed population of Y/T KO cells expressing myc-tagged YAP or empty vector (Figure S3C) or mixed population of Y/T KO cells expressing myc-tagged S94A YAP or empty vector and labeled for myc (red), CAV1 (green), and DAPI (blue; F). (G and H) Mixed cell culture of Y/T KO cells expressing either vector control or myc-tagged YAP (Figure S3D) or mixed cell culture of Y/T KO cells expressing either vector control or myc-tagged S94A YAP (H). Myc (red), CAVIN1 (green), and DAPI (blue) are shown. Arrows in (E)–(H) highlight examples of myc-YAP-expressing cells. Scale bars (E)–(H) represent 15 μm. Note, only YAP WT induces expression of CAV1 and CAVIN1. (I) Dot plot of CAV1 levels from images, as shown in (E) and (F). Each dot represents one cell. Means ± SEM. (J) Dot plot of CAVIN1 levels from images, as shown in (G) and (H). Each dot represents one cell. Means ± SEM. (K) Dot plot of CAV1 levels from mixed Y/T KO cell population expressing vector control and TAZ (WT) or TAZ (S51A) labeled for CAV1 and TAZ from images as shown in Figures S3E and S3F. (L and M) qPCR data from myc-tagged WT or S94A YAP compared to vector control expressing Y/T KO cells (L) and WT or S51A TAZ compared to vector control expressing Y/T KO cells levels (M). Means ± SD.

**Figure 3:**
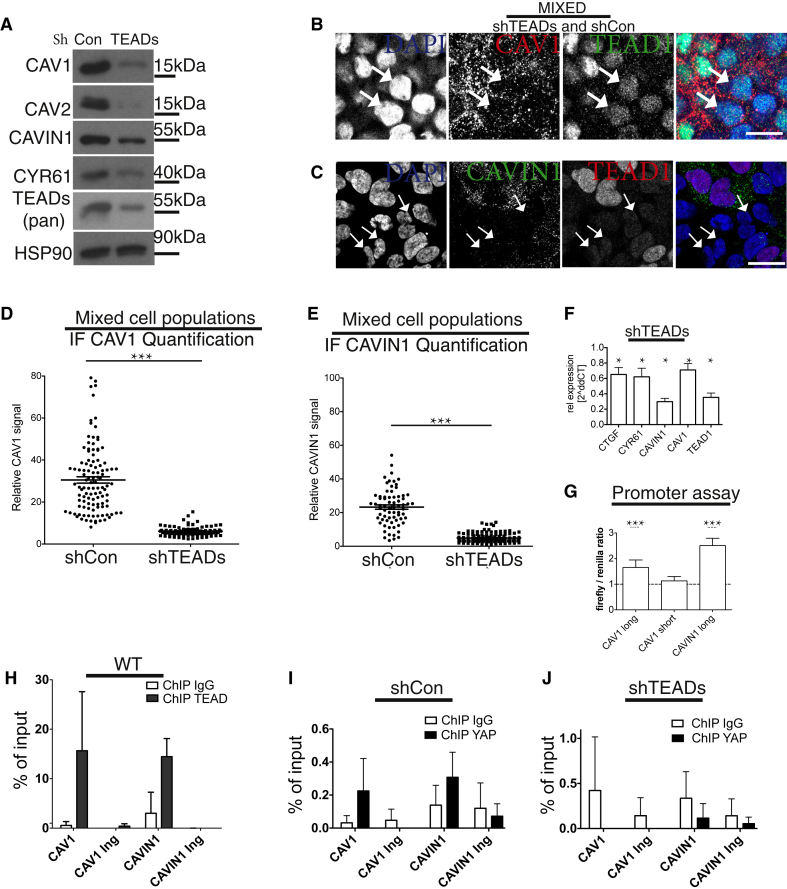
*CAV1* and *CAVIN1* Are Direct YAP/TAZ-TEAD Target Genes (A) Western blots of lysates from shTEADs and shCon HEK293A cells. (B) Mixed cell population of shTEADs and shCon HEK293A cells labeled for DAPI (blue), CAV1 (red), and TEAD1 (green). Discrete cell populations are shown in Figure S4A (see also Figure S4B). (C) Mixed cell population of shTEADs and shCon HEK293A cells labeled for DAPI (blue), TEAD1 (red), and CAVIN1 (green). Discrete cell populations are shown in Figure S4D. Arrows in (B) and (C): examples of shTEADs cells. Scale bars in (B) and (C) represent 15 μm. (D and E) Dot plot of CAV1 levels from images, as shown in (B) (Figure S4C; D) and dot plot of CAVIN1 levels from images, as shown in (C) (Figure S4E; E). Each dot represents one cell. Means ± SEM. (F) qPCR data from cells as in (A) (related to Figure S4F). Means ± SD. (G) YAP drives *CAVIN1* and *CAV1* promoter activity. Luciferase reporters were generated carrying either a short (−500 to +200 bp), or long (−1,907 to +200 bp) fragment of the *CAV1* promoter region or a fragment (−1,250 to +150 bp) of the *CAVIN1* promoter region (related to Figure S4G). The reporters were introduced into Y/T KO HEK293A cells together with either a YAP or a vector control plasmid. Note that only the long *CAV1* form contains the predicted TEAD recognition motifs. Means ± SD. (H) Real-time PCR analysis of TEAD1 chromatin immunoprecipitation (ChIP) in HEK293A cells. The precipitated DNA was quantitated using primers specific for a promoter region or a control in-gene (Ing) region. Data are means ± SD of triplicates from a representative experiment. Endogenous TEAD1 binds to both *CAV1* and *CAVIN1* promoters. (I and J) YAP ChIP for *CAV1* and *CAVIN1* in shCon HEK293A cells (I) and in shTEADs HEK293A cells (J). Endogenous YAP binds to both *CAV1* and *CAVIN1* promoters in a TEAD-dependent manner.

As YAP/TAZ are transcriptional co-activators, we explored the possibility that the essential role of YAP/TAZ in caveolar protein expression was due to transcriptional regulation. We compared mRNA levels from HEK293A Y/T KO and L1/L2 KO to WT cells (Figures 2A and 2B). In L1/L2 KO cells, with hyperactive YAP/TAZ, there was an increase in the well-established YAP/TAZ target genes *CYR61* and *CTGF* [23, 24] as well as of *CAVIN1* (Figures 2A and 2B), an effect that was paralleled by exogenously expressing hyperactive YAP (Figure S2J). Re-introduction of LATS1, but not a kinase dead version of LATS1, in L1/L2 KO cells lowered the expression of *CYR61*, *CTGF*, *CAV1*, and *CAVIN1* (Figure S2I). In addition, there was a striking absence of *CAV1* and *CAVIN1* mRNA expression as well as of *CYR61* and *CTGF* in Y/T KO cells (Figures 2A, 2B, and S2F). As YAP/TAZ bind TEAD transcription factors [24, 25, 26, 27, 28], we treated cells with verteporfin, an inhibitor of the YAP-TEAD interaction [29]. This resulted in a diminished expression of *CYR61*, *CTGF*, *CAV1*, and *CAVIN1* (Figure 2C). The binding of YAP to TEAD is partly formed via critical hydrogen bonds to serine94 in YAP [24, 25, 26]. We stably expressed either vector, WT, or S94A YAP into Y/T KO cells and assessed caveolar protein expression in these cells (Figure 2D). Only WT YAP was able to induce expression of CYR61, CAV1, and CAVIN1 (Figure 2D). We again utilized the IF-based mixed culture assay, which revealed that CAV1 and CAVIN1 expression was restored in Y/T KO cells in a cell-intrinsic manner by the re-expression of WT YAP, but not by expression of the TEAD-binding-deficient S94A YAP (Figures 2E–2J). To establish whether this was indeed due to transcriptional regulation, we performed qPCR analysis of samples prepared from Y/T KO cells expressing WT or S94A YAP. There was a significant upregulation of *CAV1* and *CAVIN1* expression upon re-expression of WT YAP (Figure 2L). To assess whether TAZ was similarly capable of inducing CAV1 and CAVIN1 expression in Y/T KO cells, we introduced either WT TAZ or a TEAD-binding-deficient TAZ mutant (S51A). Similar to YAP, exogenous TAZ expression induced CAV1 and CAVIN1 expression in the Y/T KO background in a cell-intrinsic and TEAD-binding-dependent manner (Figures 2K, 2M, S3E, and S3F). This apparent dependence on YAP/TAZ-TEAD interaction prompted us to analyze the role of TEADs in the expression of CAV1 and CAVIN1. We therefore established cells with short hairpin (sh)-mediated knockdown of TEADs. A clear TEAD dependence was evident for the expression of CAV1 and CAVIN1 (Figures 3A–3F and S4A–S4F).

To determine whether TEAD proteins can localize to *CAVIN1* and *CAV1* promoters, we examined their proximal genomic regions for TEAD recognition motifs (Figure S4G). When a genomic region of *CAV1* (−1,907 to +200 bp) or *CAVIN1* (−1,250 to +150 bp) was inserted into a plasmid upstream of a luciferase open reading frame, expression of YAP increased luciferase activity (Figure 3G). In contrast, a short *CAV1* genomic region that does not harbor TEAD recognition motifs (Figure S4G) had no effect on the luciferase activity (Figure 3G).

To examine whether this interaction also takes place *in vivo*, we carried out chromatin immunoprecipitation (ChIP) assays with antibodies against TEAD1. Regions of both *CAVIN1* and *CAV1* proximal promoters, which contain the consensus binding sequence for the YAP/TAZ-TEAD transcriptional complex, were enriched by antibodies targeting TEAD1 (Figure 3H). To test whether YAP also localized to these regions, we carried out YAP-ChIP assays in both WT and shTEAD cells. In a TEAD-dependent manner, regions in both the *CAVIN1* and *CAV1* promoters were enriched by antibodies targeting YAP (Figures 3I and 3J). These experiments indicate that the TEAD-YAP complex physically binds to promoters of both essential caveolar genes, *CAVIN1* and *CAV1*.

**Figure 4:**
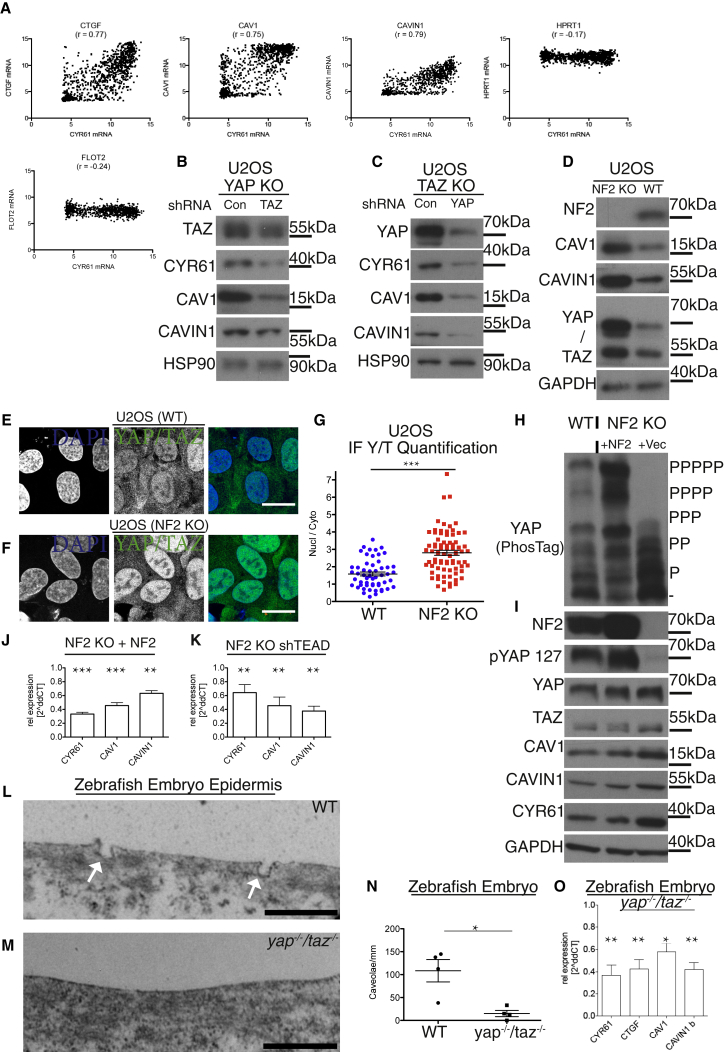
YAP/TAZ Activity Dictates Caveolar Protein Levels (A) Dot plots of *in silico* bioinformatics analysis of data obtained from cancer cell line encyclopedia (CCLE) in cell lines from 967 subjects. The analysis revealed strong positive correlation between established YAP/TAZ target genes (*CYR61* and *CTGF*) as well as between *CYR61* and *CAV1* or *CAVIN1*. However, there was no correlation for *HPRT1* or for another endolysosomal gene, such as *FLOTILLIN2* (*FLO2*), serving as negative controls (Figure S5A). R values were calculated for each correlation; p < 0.05; Pearson’s correlation coefficient. (B) Western blots from YAP KO U2OS cells with stable shRNA-induced knockdown of *TAZ* or control (Figure S5D). (C) Western blots from TAZ KO U2OS cells with stable knockdown of *YAP* or control (Figure S5D). (D) Western blots from NF2 KO and WT U2OS cells (Figure S5B). (E and F) Confocal image of U2OS WT (E) and NF2 KO cells labeled for YAP/TAZ (green) and DAPI (blue; F). Scale bars represent 20 μm. (G) Dot blots of nuclear-to-cytoplasmic ratio of YAP/TAZ, quantified of images as in (E) and (F). Each dot represents one cell. Means ± SEM. (H) Cell lysates from WT, NF2 KO cells exogenously re-expressing NF2, or NF2 KO control cells were separated in a PhosTag gel and probed for YAP. (I) Lysates, as in (H), separated on a conventional SDS-gel and processed for western blotting. (J) qPCR data from NF2 re-expressors, relative to NF2 KO U2OS control cells. NF2 re-expression reduces expression of *CYR61*, *CAV1*, and *CAVIN1*. Means ± SD. (K) qPCR data from NF2 KO U2OS shTEADs relative to control cells. *TEAD* knockdown reduces expression of *CYR61*, *CAV1*, and *CAVIN1*. Means ± SD (Figures S5B and S5C). (L) Micrographs from epidermis cells from WT zebrafish embryos. Arrows: examples of caveolae at the plasma membrane. (M) Micrographs from epidermis cells from mutant (*yap*^*−/−*^*:taz*^*−/−*^) zebrafish embryos show no caveolae. Scale bars in (L) and (M) represent 500 nm. (N) Quantification of caveolae from micrographs; each dot represents data from one fish embryo. Means ± SEM. (O) qPCR analysis of zebrafish from 5 embryos of each genotype. Means ± SD.

To explore whether regulation of *CAVIN1* and *CAV1* expression by YAP/TAZ-TEAD is a general phenomenon, we used data from cancer cell line encyclopedia (CCLE) [30] and conducted pairwise bioinformatic analysis of *CYR61* and *CTGF*, *CAVIN1*, or *CAV1* expression, respectively (Figure 4A). A strong positive correlation between the well-established YAP/TAZ-TEAD target genes *CTGF*/*CYR61* as well as for *CAV1*/*CYR61* and *CAVIN1*/*CYR61* was apparent. However, there was no correlation between expression of *CYR61* and *FLOTILLIN2* (*FLO2*), another plasma membrane protein [31], or *HPRT1*, a housekeeping gene (Figure 4A).

These data show that YAP/TAZ regulate caveolar genes and induce TEAD-mediated *CAV1* and *CAVIN1* gene expression and that *CAV1* and *CAVIN1* are direct transcriptional targets of this complex.

### YAP/TAZ Activity Dictates Caveolar Protein Levels

To investigate whether the YAP/TAZ-TEAD transcriptional complex is essential for the expression of CAV1 and CAVIN1 in other cells, we chose the osteosarcoma cell line U2OS. U2OS cells express high levels of the YAP/TAZ-TEAD target genes *CTGF* and *CYR61* as well as high levels of *CAV1* and *CAVIN1* (Figure S5A). Importantly, although YAP and TAZ are predominantly nuclear in unstimulated U2OS cells, they do have a functional Hippo pathway [23]. We attempted generating YAP/TAZ KO U2OS cells but were unsuccessful, potentially due to essential YAP/TAZ roles for cell survival in this cancer cell line. We therefore generated genome-edited single YAP and TAZ KO cell lines (Figure S5D) with either knockdown of TAZ (shTAZ) or of YAP (shYAP) (Figures 4B and 4C). In U2OS cells, as in HEK293A cells, loss of YAP and TAZ resulted in loss of CAV1 and CAVIN1 expression (Figures 4B, 4C, and S5B).

Neurofibromatosis type 2 (NF2) (also known as merlin) [1, 2, 3, 4] is the most common mutated Hippo pathway tumor suppressor gene. NF2 acts as an upstream activator of the kinase cascade and therefore as an inhibitor of YAP/TAZ [6, 7]. We deleted NF2 by CRISPR genome editing in U2OS cells to investigate whether this would increase CAVIN1 and CAV1 expression. As predicted, this was indeed the case (Figure 4D). Analyzing NF2 KO cells by IF, it was evident that YAP/TAZ localization was increased in the nucleus compared to WT U2OS cells (Figures 4E–4G). Re-introduction of NF2 into NF2-deficient U2OS cells rescued the phosphorylation state and thus activity of YAP (Figures 4H and 4I), as well as the mRNA expression and protein levels of CYR61, CAVIN1, and CAV1 (Figures 4I and 4J). To examine whether this transcriptional regulation was mediated via TEADs, we generated shTEAD, shYAP, and shTAZ NF2 KO U2OS cell lines and compared those to controls (Figures 4K and S5B). Consistent with previous results, the expression of CAV1 and CAVIN1 was also dependent on TEADs in U2OS WT cells (Figures S5B and S5C). These results demonstrate that TEADs are required for expression of CAV1 and CAVIN1 in U2OS as in HEK293A cells.

YAP/TAZ double KO is embryonic lethal in both mice [32, 33] and zebrafish [34]. To explore whether YAP/TAZ regulation of caveolar proteins is conserved across species and within cells of a living organism, we analyzed 12-somite-stage Y/T KO zebrafish as well as age- and background-matched WT embryos. The samples were processed for electron microscopy (EM), and we quantified the abundance of epidermal caveolae. There was a striking decrease in the abundance of epidermal caveolae in Y/T KO embryos (Figures 4L–4N). To examine whether this lack of caveolae was due to decreased transcriptional activity of YAP/TAZ target genes, we examined the expression levels of *cavin1* and *cav1* as well as of *cyr61* and *ctgf* in whole fish embryos (Figure 4O) and observed a substantial downregulation of all four genes in Y/T mutant embryos. Cumulatively, these data suggest that YAP/TAZ-TEAD activity is broadly critical for caveolae expression within vertebrates.

### CAVIN1 and CAV1 Regulate YAP/TAZ Activity

**Figure 5:**
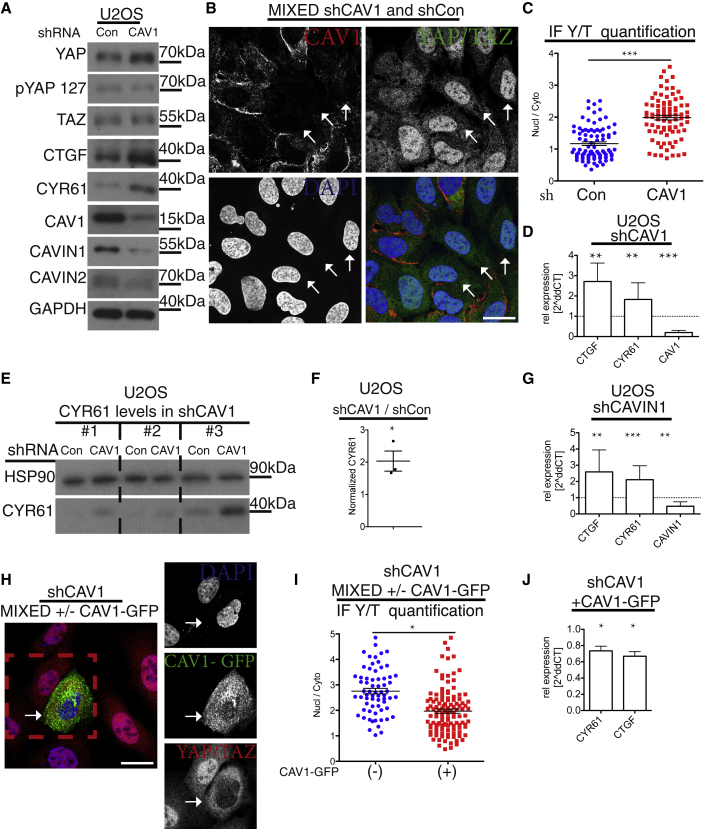
CAV1 Is a Negative Regulator of YAP/TAZ (A) Western blot analysis of shRNA-induced *CAV1* knockdown or control U2OS cells. Note higher expression of CTGF and CYR61 in *CAV1* knockdown cells. (B) Confocal image of mixed cell population of shCAV1 and shCon cells labeled for YAP/TAZ (green), CAV1 (red), and DAPI (blue; Figures S6A and S6B). Arrows: examples of shCAV1 cells. Scale bar represents 20 μm. (C) Dot plots of quantification of nuclear-to-cytoplasmic ratio of YAP/TAZ from images as in (B). Each dot represents one cell. Means ± SEM. (D) qPCR analysis of shCAV1 cells. Means ± SD. (E) Western blots from U2OS shCon and shCAV1 cell lysates from three independent experiments (nos. 1–3). (F) Quantification from blots in (E) of normalized CYR61 values in U2OS shCAV1 cells compared to shCon cells. Student’s t test. Means ± SEM; shCAV1 cells have higher levels of CYR61 than control cells. (G) qPCR analysis of shCAVIN1 U2OS cells compared to control. Means ± SD. (H) Confocal image of mixed cell population of shCAV1 (empty vector) and shCAV1 (CAV1-GFP re-expressor) cells, labeled for YAP/TAZ (red), GFP (green), and DAPI (blue). Re-expression of CAV1 in shCAV1 cells rescues hyperactivation of YAP (related to Figures S5E, S6D, S6E, S6I, and S7D–S7K). Scale bar represents 10 μm. (I) Dot plot of quantification of nuclear-to-cytoplasmic ratio of YAP/TAZ from images as in (E). Each dot represents one cell. Means ± SEM. (J) qPCR analysis of CAV1-GFP-expressing shCAV1 cell line compared to control shCAV1. Means ± SD. Further related to this figure are Figures S5G and S5F.

Although understanding of the external inputs and cell surface components for the Hippo pathway is incomplete, in many contexts, Hippo signaling and its effectors are under feedback control [7, 35]. With this premise and having established that Hippo signaling directly regulates caveolar gene expression, we investigated whether caveolae modulate the Hippo pathway. We initially generated CAV1 knockdown U2OS cells. These cells showed an upregulation of CTGF and CYR61 (Figures 5A, 5D, and 5E) protein expression as well as a decrease in the inhibitory pYAP-S127-phosphorylation (Figure 5A). The lowered protein levels of the cavin proteins (CAVIN1 and CAVIN2), upon knockdown of CAV1, is most likely due to diminished stability of cavin proteins in CAV1-deficient cells (Figures 5A, S6A, and S6B), consistent with previous reports [18]. To investigate whether the hyperactivity of YAP/TAZ was due to paracrine effects, we generated mixed cell cultures of shCAV1 and control cells and processed them in parallel for IF. Using antibodies against both CAV1 and YAP/TAZ allowed us to discriminate between these populations of cells (Figures 5B and S6B). YAP/TAZ nuclear localization was more pronounced in cells deficient of CAV1 (Figures 5B and 5C). This shows that the shCAV1-mediated YAP/TAZ hyperactivity is due to cell-intrinsic events.

To investigate whether the upregulation of *CYR61* and *CTGF* in caveolae-deficient cells was caused by increased YAP/TAZ-TEAD transcriptional activity, we prepared qPCR samples of both shCAV1 and shCAVIN1 cells and compared those to control cells (Figures 5D and 5G). In both shCAV1 and shCAVIN1 cells, an upregulation of YAP/TAZ-TEAD transcriptional activity was evident. Importantly, nuclear-to-cytoplasmic ratio of YAP/TAZ, as well as their transcriptional activity, was restored to WT levels upon re-expression of CAV1 (Figures 5H–5J and S5E). Together, these data indicate caveolar impact on YAP/TAZ-TEAD activity.

**Figure 6:**
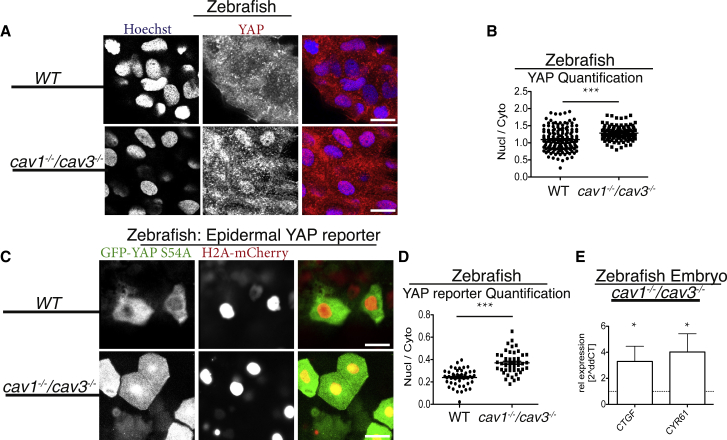
CAVEOLINs Are Negative Regulators of YAP/TAZ *In Vivo* (A) Images of epithelial cells from WT and *cav1*^*−/−*^*/cav3*^*−/*−^ 48 hpf zebrafish embryos labeled for Hoechst (blue) and YAP (red). Note the increased nuclear YAP localization in *cav1*^*−/−*^*/cav3*^*−/*−^ cells. Scale bars represent 25 μm. (B) Quantification of cellular nuclear-to-cytoplasmic ratio from *cav1*^*−/−*^*/cav3*^*−/*−^ superficial epidermal cells compared to WT. Each dot represents one cell. n > 100. Means ± SEM. (C) Images from *cav1*^*−/−*^*/cav3*^*−/*−^ and WT zebrafish embryos expressing epidermal EGFP-yap S54A (Tead-binding-deficient zebrafish yap mutant) and H2A-mCherry (nuclear marker). Note the predominantly nuclear YAP localization in the *cav1*^*−/−*^*/cav3*^*−/*−^ epidermal cells compared to WT. Scale bars represent 20 μm. (D) Quantification of nuclear-to-cytoplasmic ratio from *cav1*^*−/−*^*/cav3*^*−/*−^ epidermal cells compared to WT. Each dot represents one cell. n > 50 from each genotype. Means ± SEM (related to Figure S5H). (E) qPCR analysis from four-day-old *cav1*^*−/−*^*/cav3*^*−/*−^ and WT zebrafish embryos. Means ± SD.

To probe further the concept of caveolae as regulators of YAP/TAZ activity, we examined HEK293A cells with knockdown of *CAVIN1* and *CAV1*. Lowering either CAV1 or CAVIN1 expression caused markedly increased levels of CYR61 expression (Figure S5F). We explored whether this increase was due to transcriptional upregulation and identified that YAP/TAZ target genes indeed were increased in cells with lowered CAV1 expression (Figure S5G), as was the case for U2OS cells (Figures 5D and 5F). This suggests caveolae as an upstream negative regulator of YAP/TAZ. To examine whether this function is conserved *in vivo*, we utilized *cav1*^*−/−*^*;cav3*^*−/−*^ mutant zebrafish, which lack caveolae [12]. We developed a labeling procedure that allowed us to examine YAP localization in the epidermis of 48 hr post-fertilization (hpf) embryos (Figure 6A). There was a clear increase in the YAP nuclear-to-cytoplasmic ratio in *cav1*^*−/−*^*;cav3*^*−/−*^ mutant embryos compared to WT (Figures 6A and 6B). As YAP/TAZ mutant embryos do not survive until 48 hpf [34] and we therefore could not fully verify our YAP antibody specificity at this developmental stage, we furthermore utilized a GFP-tagged, TEAD-binding-deficient YAP construct (GFP-YAP-S54A), expressed from an epithelial promoter, and introduced it into *cav1*^*−/−*^*;cav3*^*−/−*^ mutant and WT embryos. This YAP mutant construct was co-expressed with H2A-mCherrry to allow for visualization of the nucleus (Figure 6C). In the fish embryos, we measured a significant increase in YAP nuclear to cytoplasmic localization in *cav1*^*−/−*^*;cav3*^*−/−*^ compared to WT (Figures 6C and 6D). Moreover, we examined whether the human TEAD-binding-deficient YAP counterpart (YAP-S94A) was sensitive to well-known YAP stimuli, such as serum stimulation [23], which was indeed the case (Figure S5H). The co-expression of GFP-YAP-S54A and H2A-mCherry in the embryos therefore allowed us to validate our endogenous embryonic YAP labeling data (Figures 6A–6D). In addition, embryos from *cav1*^*−/−*^*;cav3*^*−/−*^ mutants were compared to WT and we observed a marked increase in *cyr61* and *ctgf* expression (Figure 6E). Cumulatively, these data demonstrate YAP/TAZ-TEAD activity is augmented upon caveolae deficiency, and this effect is conserved across species and *in vivo*.

### Caveolae Facilitate YAP/TAZ-Mediated Flow Response

**Figure 7:**
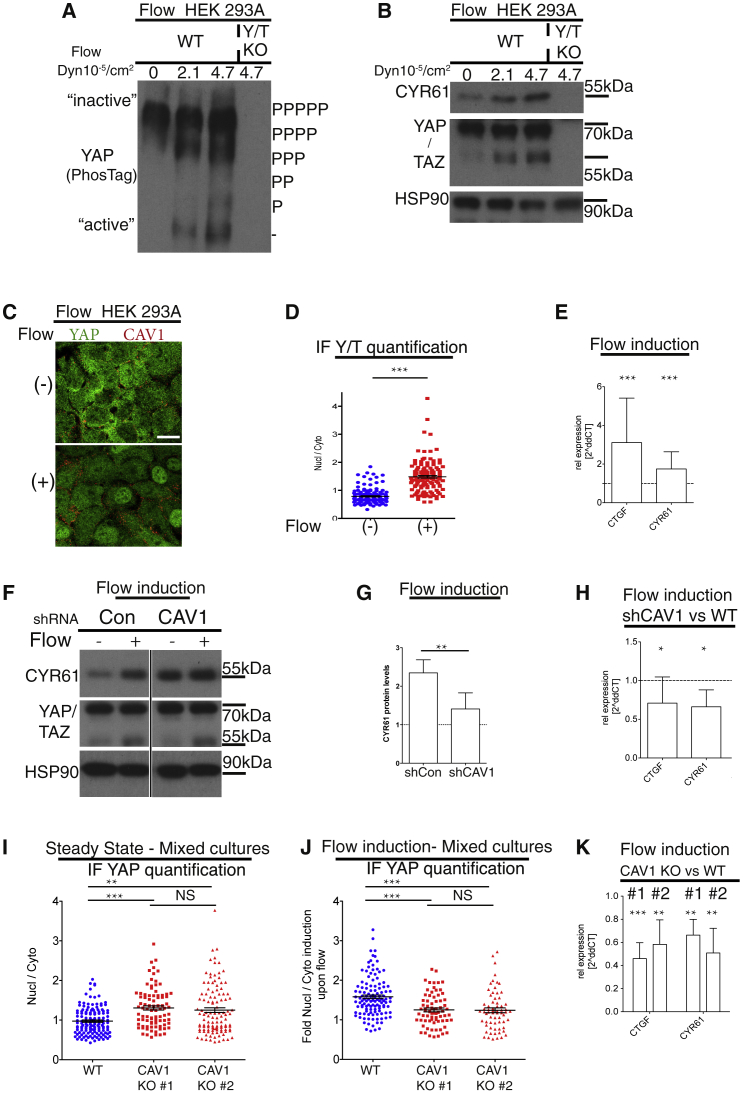
Caveolae Facilitate YAP/TAZ-Mediated Flow Response (A) Confluent HEK293A cells were kept in chambers with different flow speeds for 18 hr. YAP/TAZ KO cells were kept at high flow only. Western blot of cell lysates separated on a PhosTag gel and probed for YAP is shown. Note the dephosphorylation (downshift of YAP) and therefore predicted activation of YAP upon flow. (B) Western blots of cell lysates as in (A). Note the increase in CYR61 protein upon flow in WT cells. (C) Confocal images of cells grown without (−) or with flow at 2.1 × 10^−5^ Dyn/cm^2^ (+). Cells labeled for YAP (green) and CAV1 (red) are shown. Scale bar represents 20 μm. (D) Dot plot representing quantification of nuclear-to-cytoplasmic ratio of YAP/TAZ localization from images as in (C). Each dot represents one cell. Means ± SEM. (E) qPCR analysis of HEK293A WT cells kept at either 0 or 2.1 × 10^−5^ Dyn/cm^2^. *CTGF* and *CYR61* mRNA were increased upon flow. Means ± SD. (F) Western blot of cell lysates from either control or shCAV1 cells kept at either 0 (− flow) or at 2.1 × 10^−5^ Dyn/cm^2^ (+ flow). Note induction of CYR61 upon flow in WT cells (related to Figures S5F, S5G, and S6F). (G) Quantification of CYR61 levels from western blots as in (F), normalized to HSP90 levels. n = 3; means ± SD. (H) Relative fold induction of target gene expression in shCAV1 upon flow compared to control cells. Means ± SD. (I) Dot plots of YAP localization from images of cells at steady state. Each dot represents one cell. Means ± SEM. (J) Dot plots of normalized values of flow-induced (2.1 × 10^−5^ Dyn/cm^2^) YAP nuclear localization. Each dot represents one cell. Means ± SEM. (K) Relative fold induction of target gene expression in two separate CAV1 KO clones upon flow was compared to control cells. Means ± SD. Flow-mediated induction of *CTGF* and *CYR61* is CAV1 dependent. Further related to this figure are Figures S6G, S6H, and S7A–S7C.

As both caveolae [9] and the Hippo pathway [36, 37, 38] mediate cellular responses from shear stress, we set out to examine whether caveolae and the Hippo pathway are mechanistically linked in this process. We prepared cell lysates from confluent WT HEK293A cells grown under a range of constant flow rates (0–4.7 × 10^−5^ Dyn/cm^2^) for 18 hr and analyzed YAP/TAZ activity. Specifically, we measured the phosphorylation status of YAP and the expression of CYR61 and compared these cells to Y/T KO cells grown under shear stress. We identified a flow-dependent increase in YAP/TAZ activity, as noted by the dephosphorylation of YAP (Figure 7A), and increased protein expression of TAZ and CYR61 (Figure 7B). As 2.1 × 10^−5^ Dyn/cm^2^ is sufficient to activate YAP/TAZ (Figures 7A and 7B), and well within the range of physiological shear stress [39, 40], we chose this level for the remaining shear stress experiments. As expected, YAP nuclear localization (Figures 7C and 7D) and transcription of *CYR61* and *CTGF* were increased in cells that had experienced shear stress (Figure 7E). The induction of the YAP/TAZ target *CYR61* upon flow is therefore a suitable assay to study the shear-stress-mediated regulation of the Hippo pathway. To examine whether the activation of YAP/TAZ upon flow is mediated via caveolae, we asked whether the flow-mediated induction was different between WT cells and cells with reduced CAV1 expression levels. It was apparent that the fold induction of CYR61 protein expression upon flow was markedly reduced (by 40%) in shCAV1 cells (Figures 7F, 7G, and S6F). Indeed, in cells with efficient reduction of CAV1, the shear-stress-mediated induction of YAP/TAZ activity was diminished, as determined by the mRNA levels of *CTGF* and *CYR61* (Figure 7H). To further gain insights into the underlying mechanisms and establish whether this process was mediated via paracrine factors, we generated CAV1 KO cells (Figures S6G, S6H, and S7A–S7C). By a mixed cell culture assay, we determined the localization of YAP in CAV1 KO and WT cells. YAP was more nuclear at steady state in CAV1 KO cells (Figure 7I), and the flow-induced nuclear translocation of YAP was decreased in CAV1 KO cells compared to WT cells (Figure 7J). Furthermore, shear stress induced an increase of the YAP/TAZ-TEAD target genes *CTGF* and *CYR61* to a higher extent in WT compared to the CAV1 KO cells (Figure 7G). Importantly, the re-introduction of CAV1 into CAV1 KO cells rescued this phenotype (Figures S6I and S7D–S7M). CAV1 deficiency in L1/L2 KO did not further increase YAP/TAZ activity levels (Figures S7N–S7R), consistent with saturated YAP/TAZ activity in L1/L2 KO cells (Figures 1C–1G, S1F, and S2E) [6, 7]. These data show that YAP/TAZ are activated upon shear stress, that YAP/TAZ are hyperactivated in cells without caveolae, and that shear-stress-mediated activation of YAP/TAZ is facilitated by caveolae.

## Discussion

The Hippo pathway is recognized as critical for multiple cellular, developmental, and homeostatic processes [1, 2, 3, 4, 5]. Due to its central role in many biological contexts, tight regulation of this pathway is required and a variety of feedback mechanisms have evolved [7, 35]. In this study, we find reciprocal relationships between caveolar function and Hippo signaling. Specifically, we show that YAP/TAZ-TEAD activity directly regulates transcription of components that comprise caveolae. Conversely, loss of caveolae results in Hippo kinase cascade inactivation and thus increased YAP/TAZ-TEAD activity.

Flow sensing plays major roles in multiple cell types, especially during development [39, 40], in the cardiovascular system [40], in the kidney [39], and elsewhere [41]. The transduction of shear stress into cells has previously been shown to depend, at least in part, on caveolae [9, 11]. Still, hitherto how caveolae mediate flow sensing and other types of mechanotransduction to the interior of the cell is not well understood [9, 10, 11, 12, 13, 14]. Related, shear stress and other mechanical perturbations can affect Hippo signaling [8, 36, 37, 38]. Mechanistic insights into how the Hippo pathway is regulated by shear stress and how this cellular response is conferred has been limited [36, 37, 38]. We observed an upregulation of the matricellular remodeling factors CTGF and CYR61 with increased fluid flow. Notably, we identified that the induction of these YAP/TAZ-TEAD-dependent target genes [24] by shear stress is partly caveolae dependent. Although a complete understanding of this process remains to be determined, we show that shear stress transduction mediated by caveolae is regulated via LATS-mediated inhibitory phosphorylation of YAP/TAZ. Upon shear stress, the caveolae-facilitated activation of the Hippo kinase cascade is blunted. Consequently, caveolae-depleted cells are less sensitive to shear-stress-mediated YAP/TAZ activation. As caveolae are known to buffer cells from mechanical stress [9, 10, 11, 12, 13, 14, 42], the feedforward upregulation of caveolar components by YAP/TAZ may represent a protective response.

The connection between caveolar function and Hippo signaling may extend beyond transduction of shear stress. For example, both caveolae and the Hippo pathway also play central roles in metabolic signaling [19, 22, 43, 44], regeneration [45, 46], and cancers [1, 17]. One aspect that merits highlighting is that loss-of-function mutations within the muscle-specific caveolin isoform CAV3 [47] or CAVIN1 [48] causes muscular dystrophy. The Hippo pathway is a potent regulator of muscle cells [49, 50, 51], and intriguingly, there are overlapping gene sets between those upregulated in muscular dystrophy, caused by *CAVIN1* or *CAV3* mutations, and those driven by deregulation of muscular YAP/TAZ-TEAD transcription [48, 49, 50, 51]. It might therefore be worth pursuing to examine the state of the Hippo pathway within caveolae-deficient muscular dystrophy patients. Targeting the Hippo pathway within these patients may allow for therapeutic intervention [1, 2, 3, 4, 5]. It is noteworthy that the decrease of caveolar proteins is very dramatic upon YAP/TAZ loss of function, whereas overexpression of YAP/TAZ only modestly increases *CAV1* and *CAVEOLIN1*. It thus appears that YAP/TAZ are essential but might not be sufficient to drive expression of caveolar genes in all scenarios. We anticipate that additional factors regulating nuclear YAP/TAZ-TEAD activity, such as VGLL4 [52], MRTF-SRF [53, 54], AP1 [55, 56], and SWI/SNF [57, 58], might also play a role in regulating caveolar gene expression. The direct and reciprocal link between caveolae and the Hippo pathway provided here paves the way for further explorations into the biology of these little caves and this potent signaling pathway.

## STAR★Methods

### Key Resources Table

**Table:** 

REAGENT or RESOURCE	SOURCE	IDENTIFIER
**Antibodies**		

Anti-YAP	Santa Cruz Biotechnology	Cat# sc-101199, RRID:AB_1131430
Anti-YAP	Proteintech Group	Cat# 13584-1-AP, RRID:AB_2218915
Anti-CYR61	Santa Cruz Biotechnology	Cat# sc-13100, RRID:AB_2088733
Anti-CTGF	Santa Cruz Biotechnology	Cat# sc-14939, RRID:AB_638805
Anti-GAPDH	Santa Cruz Biotechnology	Cat# sc-25778, RRID:AB_10167668
Anti-CAV1	BD Biosciences	RRID:BD610060
Anti-CAV2	BD Biosciences	RRID:BD610684
Anti-HSP90	BD Biosciences	Cat# 610418, RRID:AB_397798
Anti-panTEAD	Cell Signaling Technology	Cat# 13295, RRID:AB_2687902
Anti-YAP p127	Cell Signaling Technology	Cat# 4911S, RRID:AB_2218913
Anti-YAP p397	Cell Signaling Technology	Cat# 13619, RRID:AB_2650554)
Anti-NF2	Cell Signaling Technology	Cat# 6995S, RRID:AB_10828709
Anti-LATS1	Cell Signaling Technology	Cat# 3477, RRID:AB_2133513
Anti-LATS2	Cell Signaling Technology	Cat# 5888, RRID:AB_10835233
Anti-CAVIN1	Cell Signaling Technology	RRID:Cat# 69036
Anti-YAP	Abcam	Cat# ab52771, RRID:AB_2219141
Anti-CAVIN1	Abcam	Cat# ab48824, RRID:AB_882224
Anti-GFP	Abcam	Cat# ab6556, RRID:AB_305564
Anti-CAVIN3	Bethyl	Cat# A302-419A, RRID:AB_1907302
Anti-CAVIN2	Atlas Antibodies	Cat# HPA039325, RRID:AB_10805473
Anti-CAV1	Jørgen Vinten, University of Copenhagen	See [59]
Anti-Myc	Cell Signaling Technology	RRID:Cat# 2276
Anti-mouse IgGs	Fisher scientific	RRID:Cat#12102270
Anti-rabbit IgGs	Fisher scientific	RRID:Cat#1805935
Anti-TEAD1	BD Biosciences	Cat# 610923, RRID:AB_398238
Anti-Goat Immunoglobulins/HRP	DAKO	RRID:P044901
Anti-Rabbit Immunoglobulins/HRP	DAKO	RRID:P044801
Anti-Mouse Immunoglobulins/HRP	DAKO	RRID:P044701
Goat anti-Rabbit IgG (H+L) Cross-Adsorbed Secondary Antibody, Alexa Fluor 488	Thermo Fisher Scientific	Cat# A-11008, RRID:AB_143165
Goat anti-Rabbit IgG (H+L) Highly Cross-Adsorbed Secondary Antibody, Alexa Fluor 594	Thermo Fisher Scientific	Cat# A-11037, RRID:AB_2534095
Goat anti-Mouse IgG (H+L) Highly Cross-Adsorbed Secondary Antibody, Alexa Fluor 594	Thermo Fisher Scientific	Cat# A-11032, RRID:AB_2534091
Goat anti-Mouse IgG (H+L) Highly Cross-Adsorbed Secondary Antibody, Alexa Fluor 488	Thermo Fisher Scientific	Cat# A-11029, RRID:AB_2534088
Goat anti-Rabbit IgG (H+L) Highly Cross-Adsorbed Secondary Antibody, Alexa Fluor 633	Thermo Fisher Scientific	Cat# A-21071, RRID:AB_2535732

**Bacterial and Virus Strains**		

Dh5-alpha *E.coli*	Sonja Vermeren, University of Edinburgh	N/A
One Shot Stbl3 Chemically Competent *E. coli*	Life Technologies	C737303

**Chemicals, Peptides, and Recombinant Proteins**		

Verteporfin	Sigma-Aldrich	SML0534-5MG
Puromycin	VWR	CAYM13884
G418	Scientific Laboratory Supplies Ltd	G5013
Hygromycin B	Scientific Laboratory Supplies Ltd	30-240-CR
GeneJet DNA transfection reagent	Tebu-Bio	189SL100488
LIPOD293 DNA transfection reagent	Tebu-Bio	189SL100668
DMEM	GIBCO	21969-035
FBS, heat inactivated	GIBCO	10500-064
Glutamine	GIBCO	25030-024
Penicillin/Streptomycin	GIBCO	15140-122
Immuno Western ECL mix	millipore	WBKLS0500
Antifade	citifluor	AF1-100
DMSO	Sigma	D2650
ProLong Diamond Antifade Mountant with DAPI	Thermo Fisher Scientific	P36962
Hoechst	Thermo Fisher Scientific	3342
Formaldehyde Solution, Methanol free	Thermo Fisher Scientific	28908
Polyacrylamide	Thermo Fisher Scientific	BP1408-1

**Critical Commercial Assays**		

RNeasy plus mini kit	QIAGEN	74136
High-capacity cDNA reverse transcriptase kit	Applied biosciences	4368814
Brilliant III Ultra-fast SYBR Green QPCR Master Mix	Agilent Technologies	600883
Dual-Glo luciferase assay system	Promega	E2920
ChIP-IT Express Enzymatic kit	Active Motif Inc.	53009
DNA Clean & Concentrator-5	Zymo Research	D4003T

**Experimental Models: Cell Lines**		

HEK293A	Kun-Liang Guan lab, UCSD	N/A
HEK293T	Kun-Liang Guan lab, UCSD	N/A
U2OS	Jack Dixon, UCSD	N/A

**Experimental Models: Organisms/Strains**		

*wwtr1* mutant zebrafish	Link Lab, Medical College of Wisconsin	Allele mw49
*yap1* mutant zebrafish	Link Lab, Medical College of Wisconsin	Allele mw48
*cav1* mutant zebrafish	Poss Lab, Duke University	Allele pd1104
*cav3* mutant zebrafish	Bagnat Lab, Duke University	Allele pd1149

**Oligonucleotides**		

Please refer to Tables S1 and S2	N/A	N/A

**Recombinant DNA**		

Please refer to Table S3	N/A	N/A

**Software and Algorithms**		

Fiji	ImageJ	https://fiji.sc/
Prism	GraphPad	https://www.graphpad.com/scientific-software/prism/
Photoshop and InDesign	Adobe	https://www.adobe.com/uk/creativecloud.html
Excel and Word	Microsoft	https://www.office.com/
BioRender	BioRender	https://app.biorender.io/

**Other**		

Quasi Vivo QV500 system	Kirkstall	QV500
PF22X0103 peristaltic pump	Parker	PARKER PF22X0103
Thermanox 13mm coverslips	Thermo Fisher Scientific	#174950
LightCycler 96 System	Roche	N/A
X Ray Film 18x24cm Double Sided	Scientific Laboratory Supplies Ltd	MOL7016
ThermanoxTM 13 mm coverslips	Thermo Fisher Scientific	#174950
COVER GLASS ROUND Ø 13 MM NO.1	VWR International Ltd.	631-1578
Immobilon(R)-P Polyvinylidene difluoride membranes,size 26.5 cm x 375 cm	Sigma-Aldrich	P2938-1ROL

### Contact for Reagent and Resource Sharing

Further information and requests for resources and reagents should be directed to and will be fulfilled by the Lead Contact, Carsten G. Hansen (Carsten.g.hansen@ed.ac.uk).

### Experimental Model and Subject Details

#### *In vitro* studies: cell lines

All cell lines were cultivated at 37°C in a humidified, 5% CO_2_ atmosphere. HEK293A, HEK293T, and U2OS cells were cultivated in high glucose DMEM (GIBCO) supplemented with penicillin, streptomycin, 2 mM glutamine, and 10% fetal bovine serum (FBS) if nothing else stated.

The HEK293A and HEK293T cell line are human epithelial cells originating from embryonic kidney of a female. The wild-types (WT) as well as the HEK293A YAP/TAZ, and LATS1/2 double knockout cell lines [22, 35] were kindly provided by Professor Kun-Liang Guan lab, University of California, San Diego (UCSD). HEK293A cell lines were used to perform experiments while HEK293T cells where used for virus generation. The U2OS cell line originates from human epithelial from the bone of a female suffering from osteosarcoma. The U2OS wild-type was kindly provided to us by Professor Jack Dixon (UCSD). Cells were periodically tested for Mycoplasma contamination but were not authenticated.

#### *In vivo* studies: zebrafish

*Danio rerio* (ZDR strain) were raised and housed in a multi-rack Pentaire aquatics system using water purified by high capacity reverse osmosis coupled with mechanical and biological filtration. The room maintained at 28.5°C and under a 14 hr light / 10 hr dark cycle. Zebrafish were not immunized, but the colony was routinely screened for the following pathogens. All specimens were negative the pathogens assayed: Edwardsiella ictaluri, Flavobacterium columnare, Ichthyophthirius multifiliis, Infectious spleen & kidney necrosis virus (ISKNV), Mycobacterium spp., Mycobacterium abscessus, Mycobacterium chelonae, Mycobacterium fortuitum, Mycobacterium haemophilum, Mycobacterium marinum, Mycobacterium peregrinum, Myxidium streisingeri, Piscinoodinium pillulare, Pleistophora hyphessobryconis, Pseudocapillaria tomentosa, Pseudoloma neurophilia The embryos were harvested at early developmental stages (at the latest 48 hpf) and were not sex matched. Subjects were naive and not previously involved in other procedures prior to the experiments reported here.

The following lines were used to generate *yap;taz* (*wwtr1*) double homozygous mutants.

*yap1*^mw48^ (c. 158-161del). This allele has a 4 bp deletion within exon1 [34].
*wwtr1*^mw49^ (c. 156-160del). This allele has a 5 bp deletion within exon1 [34].

In addition, *cav1;cav3* double homozygous mutants have been described elsewhere [12]. All experiments involving zebrafish were performed in compliance with the Institutional Animal Care and Use Committee of the Medical College of Wisconsin, protocol number AUA1378.

### Method Details

#### Generation of knockdown (KD), knockout (KO), and re-expression cell lines

KD cell lines were generated by retrovirus mediated shRNA knockdown. The virus was generated by co-transfecting pMD2G and pSPAX2 (from the Kun-Liang Guan lab, UCSD) with pKLO.1-vectors coding for shRNAs mediated targeting of YAP (TRCN0000300325), TAZ (TRCN0000370007), and TEADs 1/3/4 as previously described [22, 24], or CAV1 TRCN0000008002 (shCAV1 #1), TRCN0000007999 (shCAV1 #2), or CAVIN1 (TRCN0000430242). shRNA constructs are listed in are listed in Table S3. The virus was harvested, filtered and added to polybrene treated cells of interest. Following 6-8 hr incubation the cell medium was changed. Stable cell lines were generated following puromycin selection. To generate U2OS CAV1 re-expressing cell lines, shCAV1 cells were transfected with CAV1-GFP plasmid (#14433, Addgene) and stable exogenous CAV1-expressing cells were established by G418-selection (SLS). Plasmids encoding either WT or mutant YAP (S94A or 5SA) as well as WT or mutant TAZ (S51A) are listed in Table S3 [24, 27]. HEK293A CAV1 re-expressing cell lines were generated by lentiviral transduction of CAV1 KO cells with a pBabe vector carrying the open reading frame of human CAV1. Clonal YAP and TAZ (*WWTR1*) knock-out cell lines were generated by transient transfection of CrispR constructs, followed by two days of puromycin selection and one day recovery in medium without puromycin, followed by single cell sorting into 96-well plates [22]. To generate CrispR-mediated CAV1 KO cell lines, the following oligo nucleotides were ligated and thereafter inserted into pSpCas9(BB)-2A-Puro (PX459 *V2.0*; Addgene plasmid #48139): CAV1crispr#1fw CACCGAGTGTACGACGCGCACACCA, CAV1crispr#1rev AAACTGGTGTGCGCGTCGTACACTC and CAV1crispr#2fw CACCGTTTAGGGTCGCGGTTGACC, CAV1crispr#2rev AAACGGTCAACCGCGACCCTAAAC. Cells were transfected using LipoD293 or GenJet transfection reagent (SignaGen Laboratories). CrispR oligos are listed in Table S2.

#### Transformation

Transformation of competent bacteria was carried out by heat shock, where after bacteria were seeded onto selective Petri dishes. The following day single clones were picked and propagated for plasmid harvest.

#### Western blotting

Cell lysate preparation in reducing lysis buffer and western blotting was performed as described in Hansen et al., 2015 [22] using the following antibodies against YAP/TAZ (sc101199), CYR61 (sc13100), CTGF (sc14939), and GAPDH (sc25778) all from Santa Cruz as well as CAV1 (BD610060), CAV2 (BD610684), and HSP90 (BD610418) from BD Biosciences, pan TEAD (3295), pYAP 127 (4911), pYAP 397 (13619), NF2 (Merlin, 6995), LATS1 (3477), LATS2 (5888), and CAVIN1 (PTRF, 69036) from Cell Signaling Technology, and YAP (ab52771), CAVIN1 (PTRF, ab48824) (only used for western blotting in this study), and GFP (ab6556) from Abcam. Antibodies against CAVIN3 (SRBC, A302-419A) were from Bethyl laboratories and against CAVIN2 (SDPR, HPA039325) were from Sigma. Monoclonal antibodies raised against CAV1 were provided by Jørgen Vinten [59] (The Panum Institute, University of Copenhagen). Furthermore, HRP-conjugated secondary antibodies were used for western blotting.

Besides conventional SDS gels western blotting was performed with Phos-Tag gels. Therefore, 15 μL Phos-tag reagent (Wako chemicals) and 25 μL of 10mM MnCl_2_ were added to each 7.5% (w/v) polyacrylamide gel while the subsequent steps remained the same. Blots were developed using Immuno Western ECL mix (Millipore) and X-ray films (SLS). A larger area of the scans of western blot films is shown in Data S1.

#### RT-qPCR and Primers

For quantitative reverse transcription PCR (RT-qPCR) mRNA was extracted from cells using the RNeasy plus mini kit (QIAGEN). Complementary DNA (cDNA) was generated using High-Capacity cDNA Reverse Transcriptase kit (Applied Biosystems). qPCR was performed on 1 ng of cDNA using the Brilliant III Ultra-Fast SYBR Green QPCR Master Mix (Agilent Technologies) and the LightCycler 96 System (Roche) according to manufactures instruction. In mammalian cells expression levels of all genes analyzed were normalized to Hypoxanthine-guanine phosphoribosyltransferase (*HPRT1*) levels [22]. Primer sets against caveolar components were designed using primerbank [60]. qPCR primers, harvest of samples and preparation of cDNA from zebrafish embryos were carried by established methods [34, 42]. Samples were normalized to Elongation factor 1-alpha (*ef1a*) [34]. Primers are listed in Table S1.

#### Chromatin immunoprecipitation qPCR (ChIP-qPCR)

ChIP was performed on HEK293A WT cells using the ChIP-IT Express Enzymatic kit (Active Motif Inc.). For immunoprecipitation anti-mouse IgGs (whole molecule, #12102270, Fisher Scientific), anti-rabbit IgGs (whole molecule, #11805935, Fisher Scientific), anti-TEF-1 (TEAD1, #610923, BD Bioscience), and anti-YAP (ab52771, Abcam) antibodies were used. Subsequent DNA clean up (by DNA Clean & Concentrator-5, Zymo Reseach) was followed by DNA quantification by qPCR analysis and output was normalized to the input DNA. Primer sequences are as follows 5′-TGGCATAACCTGTTGGCATA-3′ and 5′-CCCAAACGCTTCGAAATAAG-3′ (forward and reverse) *CAV1* promoter (CAV1), 5′-TTTCAGAATCTCCTGGTCCAC-3′ and 5′-TTGCTCTCTGTTTCCCTCTC-3′ *CAVIN1* promoter (CAVIN1), 5′-CCTCCGTGTCTCAGTGGTTT-3′ and 5′-TCACCTTGCTTGCCTTTCTT-3′ *CAV1* in gene (CAV1 Ing), and 5′-GCAGTTTTGAGGAGGCAAAG-3′ and 5′-AAATGCTTCCTGGCCCTTAT-3′ *CAVIN1* in gene (CAVIN1 Ing) and *CTGF* was used as a control [22].

#### Immunofluorescence (IF) Microscopy

Cells were seeded onto poly-D-lysine (Sigma) coated coverslips, fixed with 37**°**C 4% formaldehyde (Sigma) (v/v in PBS), permeabilized and incubated in IF buffer (2.5% FBS, 0.3% Triton-x-100 (v/v) in PBS) with antibodies listed above, washed and subsequently incubated with fluorophore-conjugated secondary antibodies (Thermofisher). Cells on the coverslip were mounted on glass slides using ProLong Diamond Antifade Mountant with DAPI stain (Thermofisher). This procedure is described in more detail in [61]. Image acquisition was performed by a Zeiss 780 inverted confocal laser scanning microscope (CLSM) utilizing a Plan-Apochromat 63 × /1.4 Oil DIC M27 objective. For quantification of IF images a polygonal region of interest (ROI) was drawn manually and used to quantify the pixel mean of the signal in channels of interest. For quantification of nuclear to cytoplasmic (Nucl/Cyto) ratio the nucleus was identified in the DAPI channel and used to draw a ROI. Subsequently, the value of this ROI was measured in the channel of interest and tabulated. The ROI was then moved to the cytoplasm, measured and tabulated. Nucleoli were excluded from ROIs. For quantification of CAV1 or CAVIN1 levels, the confocal plane was selected to be at the base of the cells. ROI of the signal in these images was measured, and tabulated in Microsoft Excel, depending on the absence or presence of YAP/TAZ, TEAD or CAV1 signal, again by flicking between channels in Fiji (ImageJ). Cells undergoing mitosis or with multi nuclei, as determined by DAPI stain, were not included in the quantification. Five to 15 cells of each population, as determined by the presence or absence of signal in the other channel, were quantified from between 8 - 15 different images. The ratios were calculated in Microsoft Excel and plotted as a scatterplot in GraphPad Prism.

#### Verteporfin treatment

HEK293A WT cells were treated with 6.45 μM verteporfin (Sigma-Aldrich) with 1.87% (v/v) dimethyl sulfoxide (DMSO, Sigma-Aldrich) and compared to DMSO treated cells. After 18 hr, mRNA was extracted and RT-qPCR was performed [22].

#### Proximal promoter cloning and luciferase assay

The upstream promoter regions of *CAV1* and *CAVIN1* were examined for M-CAT and putative TEAD binding motifs. pGL3-basic vectors carrying upstream proximal promoter regions of either *CAV1* (−1907 to +200bp) or *CAVIN1* (−1250 to +150bp) were generated by restriction cloning and insertion into the multiple cloning site using Mlu1/Xho1 (*CAV1*) or Kpn1/Mlu1 (*CAVIN1*) respectively. A short version (−550 to +200bp) of the *CAV1* promoter was also generated, which does not harbor any predicted TEAD recognition sites. HEK293A YAP/TAZ KO cells were co-transfected with either one of the plasmids above and pCMV Flag-YAP. 36-48 hr after transfection cells were lysed using lysis buffer (91.5 mM K_2_HPO_4_, 8.5 mM KH_2_PO_4_, 0.2% Triton X-100 (v/v), 1 mM DTT, 1 mM PMFS) and lysates were transferred into a white 96-well plate (Corning). Luminescence was induced by using the Dual-Glo luciferase assay system (Promega) and was detected by a Biotek HT plate reader. Luciferase activity was normalized to renilla signal.

#### Flow studies

Cells were cultured under laminar flow using the Kirkstall Quasi Vivo QV500 system with a Parker (PF22X0103) peristaltic pump. Cells were seeded onto Thermanox 13 mm coverslips (#174950, ThermoFisher), or (for IF) poly-D-lysine coated glass coverslips and grown to confluency in complete DMEM containing 10% FBS. The coverslips were transferred into the QV500 system and cells were cultured in DMEM (with supplements as described above) containing 0.1% FBS and a constant flux of 4.7 and 2.1^∗^10^−5^ Dyn/cm^2^, respectively. After 18 hr cells were harvested and analyzed by RT-qPCR, western blotting or IF.

#### Zebrafish Yap localization reporter

Yap localization was assessed specifically in zebrafish epidermal cells by expression of tol2 -*4.4kb*krt18:eGFP-YapS54A. Plasmid DNA was co-injected with Tol2 transposase as well as tol2 h2ax:H2A-mCherry plasmid DNA to mark cell nuclei. The YapS54A mutant (derived from *Danio rerio*) disrupts Tead factor binding and thus prevents deleterious gain-of-function phenotypes [34]. At 48 hpf, embryos were fixed in 4% paraformaldehyde for 1 hr and then washed extensively in PBST. Embryos were then mounted in 1% agarose and imaged using a 40x water-immersion objective on a Nikon C1 scanning laser confocal microscope. Epidermal cells expressing both eGFP-YapS54A and H2A-mCherry were imaged. For quantifying pixel intensity, image slices with the maximum nuclear diameter were selected. Total fluorescence intensity was calculated for the entire cell and for just the nucleus by measuring the eGFP pixel intensity from the corresponding mCherry fluorescence. Cytoplasmic intensity was calculated by subtracting the nuclear from total cell measurements. Image acquisition and measurements were obtained in a genotype masked manner. Data presented are from 50 cells from at least 10 embryos for each genotype.

For whole mount immunohistochemistry fixed zebrafish embryos were washed in PBST and stored in 100% methanol at −20°C overnight. This was followed by rehydration in solution of incrementally increased PBST concentration (25%, 50%, 75% PBST in methanol) and a final wash in PBST. Subsequently, embryos were incubated in blocking buffer (10% goat serum (Sigma), 0.2% BSA (Sigma) in PBST) for several hours at room temperature. For labeling of YAP, embryos were left in blocking solution with anti-YAP antibody (#13584-1-AP, Proteintech) at 4°C overnight. After rigorous washing in PBST, embryos were incubated in blocking solution containing secondary antibody (Alexa Fluor 633, A21071, Thermofisher), which was followed by another excessive washing procedure. Nuclei were labeled by Hoechst 3342 (Thermofisher). Finally, embryos were dehydrated in glycerol solutions (30%, 50%, 80% glycerol/PBS) and stored in antifadent (AF1, Citifluor). Labeled zebrafish embryos were mounted in antifadent onto glass slides and microscopic images were taken using the Leica SP8 CLSM utilizing a HC PL APO 40x oil CS2 objective. A single plane image was taken for image analysis. The nuclear to cytoplasmic ratio was quantified in epidermal cells where Hoechst signal (nuclear diameter) was the widest. The nuclear to cytoplasmic YAP ratio in individual cells from at least five separate images from each genotype was plotted in Prism (GraphPad).

#### Transmission Electron Microscopy (EM)

Zebrafish embryos were collected at the 12 somite stage. Briefly, the blocks were trimmed on a Leica RM2255 microtome, and ultra-thin sections were cut and collected on coated grids, and stained with uranyl acetate and lead citrate [62]. For each embryo, twenty non-overlapping images of epidermis were captured on a Hitachi H600 at 30,000x. Caveolae within the epidermis were quantified for each micrograph in a masked manner where the individual scoring was unaware of the sample genotype. Four embryos were analyzed for each genotype.

### Quantification and Statistical Analysis

Data is represented with significance values (p) used by “^∗^” p < 0.05, “^∗∗^” p < 0.01 and “^∗∗∗^” p < 0.001. All data originated from at least three independent biological replicates if not directly stated otherwise. Data was analyzed using Fiji (ImageJ), Excel (Microsoft) and Prism (GraphPad) software. All statistical analysis and graphs were generated using Prism software. No optimal sample-size estimation was calculated. The genotype of samples imaged in Figures 4L and 4M were blinded for the person quantifying the number of caveolae. For IF based quantification random images of areas of the coverslips were captured. Cells that had multiple nuclei, were not optimal processed or were undergoing mitosis were not included. All IF based scatterplots were tested and analyzed by unpaired Student’s t test, whereas all other data was analyzed using Mann-Whitney, if not stated otherwise. Data are represented as mean ± standard error of the mean (SEM) or standard deviation (SD) as highlighted in figure legends. For correlation analysis, gene expression data across a panel of 967 cancer cell lines were downloaded from the Cancer Cell Line Encyclopedia (CCLE) [30] and the correlations between mRNA expressions of each pair of genes were evaluated by Pearson’s correlation coefficient (r) with two tailed p values, < 0.05 considered significant.

## Supplemental Information

Document S1. Figures S1–S7 and Tables S1–S3

Data S1. Full Scans of WBs Shown in the Figures

Document S2. Article plus Supplemental Information
