# Supplementary material for: The Hippo Pathway Regulates Caveolae Expression and Mediates Flow Response via Caveolae
Source: Curr Biol. 2019 Jan 21;29(2):242–255.e6. doi: 10.1016/j.cub.2018.11.066 (PMC6345631; doi:10.1016/j.cub.2018.11.066)
Supplement: Document S1. Figures S1–S7 and Tables S1–S3 [file mmc1.pdf]

Current Biology, Volume *29*

Supplemental Information

The Hippo Pathway Regulates Caveolae Expression and Mediates Flow Response via Caveolae

Valentina Rausch, Jonathan R. Bostrom, Jiwon Park, Isabel R. Bravo, Yi Feng, David C. Hay, Brian A. Link, and Carsten G. Hansen

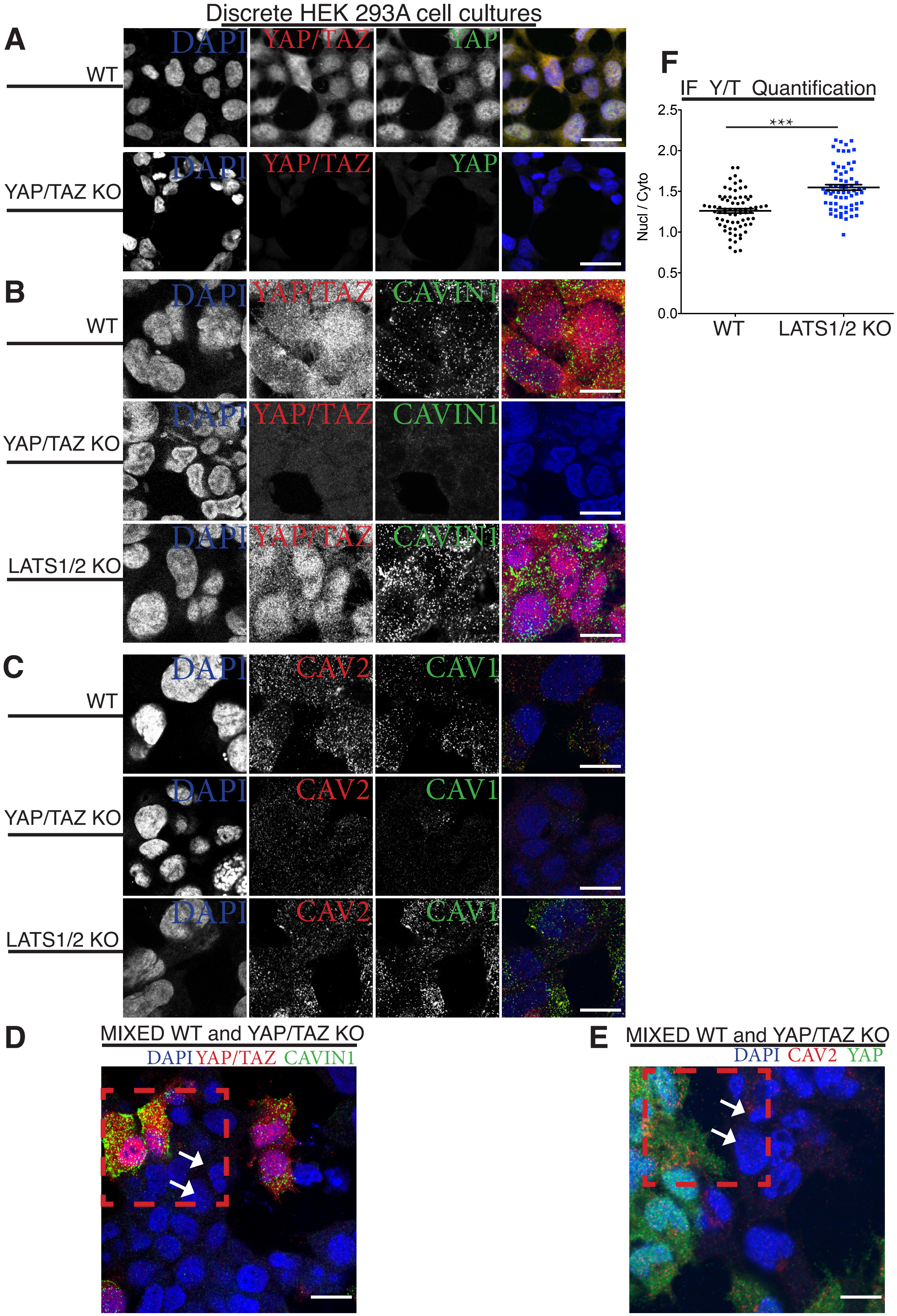

Discrete HEK 293A cell cultures
A
WT
YAP/TAZ KO
DAPI
YAP/TAZ
YAP
B
WT
YAP/TAZ KO
LATS1/2 KO
DAPI
YAP/TAZ
CAVIN1
C
WT
YAP/TAZ KO
LATS1/2 KO
DAPI
CAV2
CAV1
D
MIXED WT and YAP/TAZ KO
DAPI YAP/TAZ CAVIN1
E
MIXED WT and YAP/TAZ KO
DAPI CAV2 YAP
F
IF Y/T Quantification
Nucl / Cyto
2.5
2.0
1.5
1.0
0.5
0.0
***
WT
LATS1/2 KO

Figure S1. Validation of antibody specificity allows for single cell immunofluorescence based studies. Related to Figure 1

(A) Confocal image of discrete HEK 293A cell populations of WT and YAP/TAZ KO cells. The cells were fixed and labeled for YAP/TAZ (red), YAP (green), and counterstained for DAPI (blue). The single channels (green and red) allow visualization of YAP/TAZ and YAP across the different genotypes, and highlight the specificity of the YAP/TAZ and YAP antibodies used. Scale bar = 15μm.

(B) Discrete HEK 293A cell populations of WT, YAP/TAZ KO, and LATS1/2 KO cells labeled for YAP/TAZ (red), CAVIN1 (green), and DAPI (blue). Images like these, were used for quantification represented in dot plot in Figure 1E. Scale bar = 15μm **(C)** Discrete HEK 293A cell populations of WT, YAP/TAZ KO, and LATS1/2 KO cells labeled for CAVEOLIN2 (CAV2, red), CAV1 (green), and DAPI (blue). Scale bar = 15μm**. (D)** Zoomed out image of mixed cell culture depicted in Figure 1K, labeled for YAP/TAZ (red), CAVIN1 (green), and DAPI (Blue). Red box highlights image shown in Figure 1K. Arrows: Examples of Y/T KO cells. **(E)** Zoomed out image of mixed cell culture depicted in Figure 1M. Cells were labeled for CAVEOLIN2 (CAV2) (red), YAP (green), and DAPI (blue). Arrows: Examples of Y/T KO cells. Note cells with no YAP/TAZ signal have low CAV2 signal. Red box highlights image shown in Figure 1M. Scale bars D and E = 15μm. **(F)** Dot plot representation of quantification from discrete populations of YAP-labeled WT and LATS1/2 KO cells as in Figure S1B. Means ± SEM.

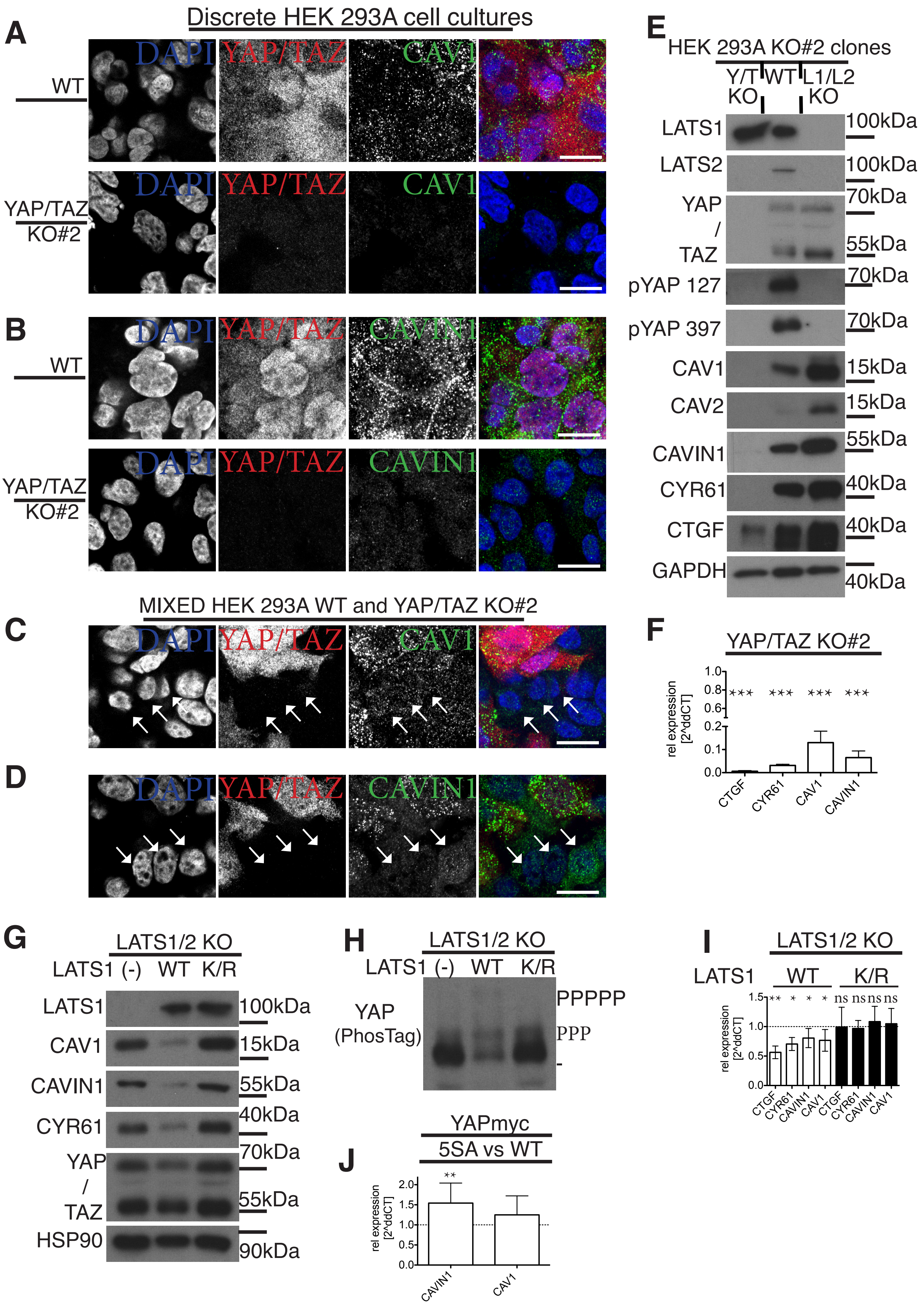
Discrete HEK 293A cell cultures
A
WT
YAP/TAZ KO#2
DAPI
YAP/TAZ
CAV1
B
CAVIN1
MIXED HEK 293A WT and YAP/TAZ KO#2
C
D
E
HEK 293A KO#2 clones
Y/T KO
WT
L1/L2 KO
LATS1
LATS2
YAP / TAZ
pYAP 127
pYAP 397
CAV1
CAV2
CAVIN1
CYR61
CTGF
GAPDH
100kDa
70kDa
55kDa
15kDa
40kDa
F
YAP/TAZ KO#2
rel expression [2^ddCT]
***
G
LATS1/2 KO
LATS1 (-) WT K/R
HSP90
90kDa
H
YAP (PhosTag)
PPPPP
PPP
-
I
ns
J
YAPmyc 5SA vs WT
**

Figure S2. YAP/TAZ are essential for the expression of CAV1 and CAVIN1 in separate gene edited clones. Related to Figure 1 and 2

(A) Confocal image of discrete HEK 293A cell populations of WT and an independently generated YAP/TAZ KO clone termed YAP/TAZ KO#2. The cells were fixed and labeled for YAP/TAZ (red), CAV1 (green), and DAPI (blue). All processing of samples for immunofluorescence and imaging was done in parallel with similar microscope settings to allow for direct comparison. Scale bars = 15μm. **(B)** Discrete HEK 293A cell populations of WT and YAP/TAZ KO#2 cells labeled for YAP/TAZ (red), CAVIN1 (green), and DAPI (blue). **(C)** Mixed cell culture of YAP/TAZ KO#2 and WT HEK 293A cells labeled for YAP/TAZ (red), CAV1 (green), and DAPI (blue). Arrows: Examples of YAP/TAZ KO#2 cells. Note cells with no YAP/TAZ signal have low CAV1 signal. Scale bar = 15μm. Images are related to Figures 1I. **(D)** Mixed cell culture of YAP/TAZ KO#2 and WT HEK 293A cells labeled for YAP/TAZ (red), CAVIN1 (green), and DAPI (blue). Arrows: Examples of YAP/TAZ KO#2 cells. Scale bar = 15μm. Images are related to Figure 1K. **(E)** Western blots as in Figure 1F but from YAP/TAZ KO#2 (Y/T KO), WT, and LATS1/2 KO#2 (L1/L2 KO) HEK 293A cells. **(F)** qPCR data from YAP/TAZ KO#2 HEK 293A cells compared to WT. Means ± SD. **(G)** Western blots of LATS1/2 KO HEK 293A cells stably expressing either vector control (-), LATS1 WT, or kinase dead (K/R) LATS1; related to Figure 1F **(H)** PhosTag-based Western blot analysis of the same lysates as in G. **(I)** qPCR analysis of LATS1/2 KO HEK 293A cell lines expressing LATS1 WT or LATS1 K/R and compared to vector control. Means ± SD. Note that only the expression of kinase active LATS1 decreases expression of established YAP/TAZ-TEAD target genes *CYR61* and *CTGF* as well as of *CAV1* and *CAVIN1* **(J)** qPCR analysis of HEK 293A cells expressing myc-tagged YAP-WT or YAP-5SA (YAP construct with all five LATS1/2 mediated phosphorylation sites mutated). Data is plotted as fold induction of *CAVIN1* and *CAV1* of YAP-5SA construct compared to YAP-WT. Means ± SD.

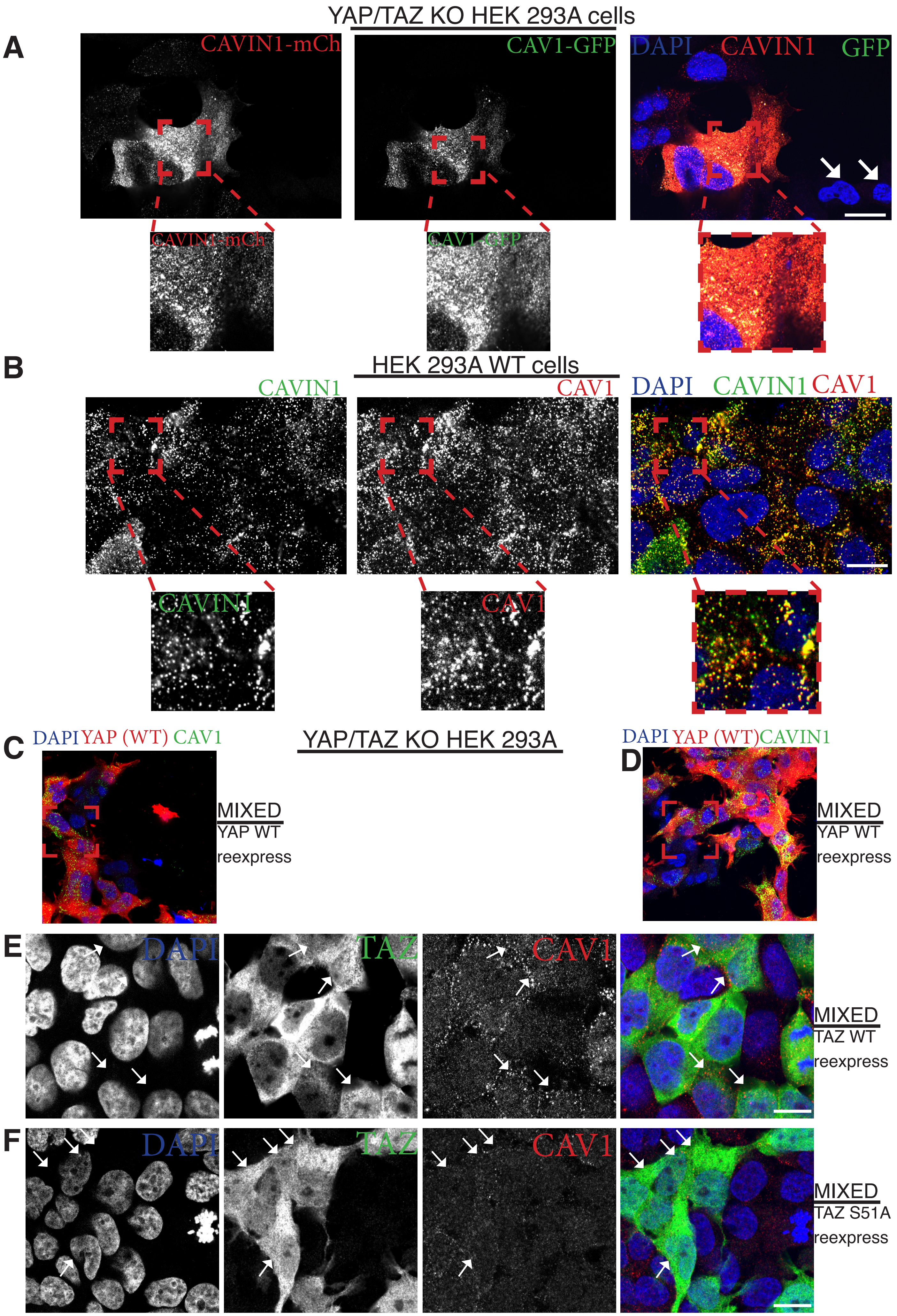

YAP/TAZ KO HEK 293A cells
A
CAVIN1-mCh
CAV1-GFP
DAPI CAVIN1 GFP
CAVIN1-mCh
CAV1-GFP
B
HEK 293A WT cells
CAVIN1
CAV1
DAPI CAVIN1 CAV1
CAVIN1
CAV1
C
DAPI YAP (WT) CAV1
YAP/TAZ KO HEK 293A
DAPI YAP (WT) CAVIN1
D
MIXED
YAP WT
reexpress
MIXED
YAP WT
reexpress
E
DAPI
TAZ
CAV1
MIXED
TAZ WT
reexpress
F
DAPI
TAZ
CAV1
MIXED
TAZ S51A
reexpress

Figure S3. Upon exogenous expression in Y/T KO cells, CAV1 and CAVEOLIN1 generate caveolae-like plasma membrane domains. Related to Figure 1 and 2

(A) YAP/TAZ KO HEK 293A cells were transfected with CAVIN1-mCherry and CAV1-GFP and processed for immunofluorescence. Note that re-expressed CAVIN1 and CAV1 co-localize in plasma membrane domains. Arrows: Untransfected YAP/TAZ KO cells. Red boxes highlight inserts shown below. Scale bar = 20μm. **(B)** HEK 293A WT cells were labeled for endogenous CAVIN1 (green), CAV1 (red), and counterstained for DAPI (blue). Red boxes highlight inserts shown below. Note CAVIN1 and CAV1 co-localize in plasma membrane domains. Scale bar = 20μm. **(C)** Zoomed out image of mixed cell culture depicted in Figure 2E. YAP/TAZ KO HEK 293A cells expressing either vector control or myc-tagged YAP. myc (red), CAV1 (green), and DAPI (blue). **(D)** Zoomed out image of mixed cell culture depicted in Figure 2G. YAP/TAZ KO HEK 293A cells expressing either vector control or myc-tagged YAP. myc (red), CAVIN1 (green), and DAPI (blue). Red box in D highlight images shown in Figure 2G. **(E)** Mixed cell culture of YAP/TAZ KO HEK 293A cells expressing either vector control or TAZ. TAZ (green), CAV1 (red), and DAPI (blue). Arrows: Cells expressing TAZ. Scale bar = 15μm**.** **(F)** Mixed cell culture of YAP/TAZ KO HEK 293A cells expressing either vector control or TEAD-binding deficient TAZ S51A. TAZ (green), CAV1 (red), and DAPI (blue). Arrows: Cells expressing mutant TAZ. Scale bar = 15μm. Images as those in E and F form the basis of the quantification of the dot plot representation in Figure 2K.

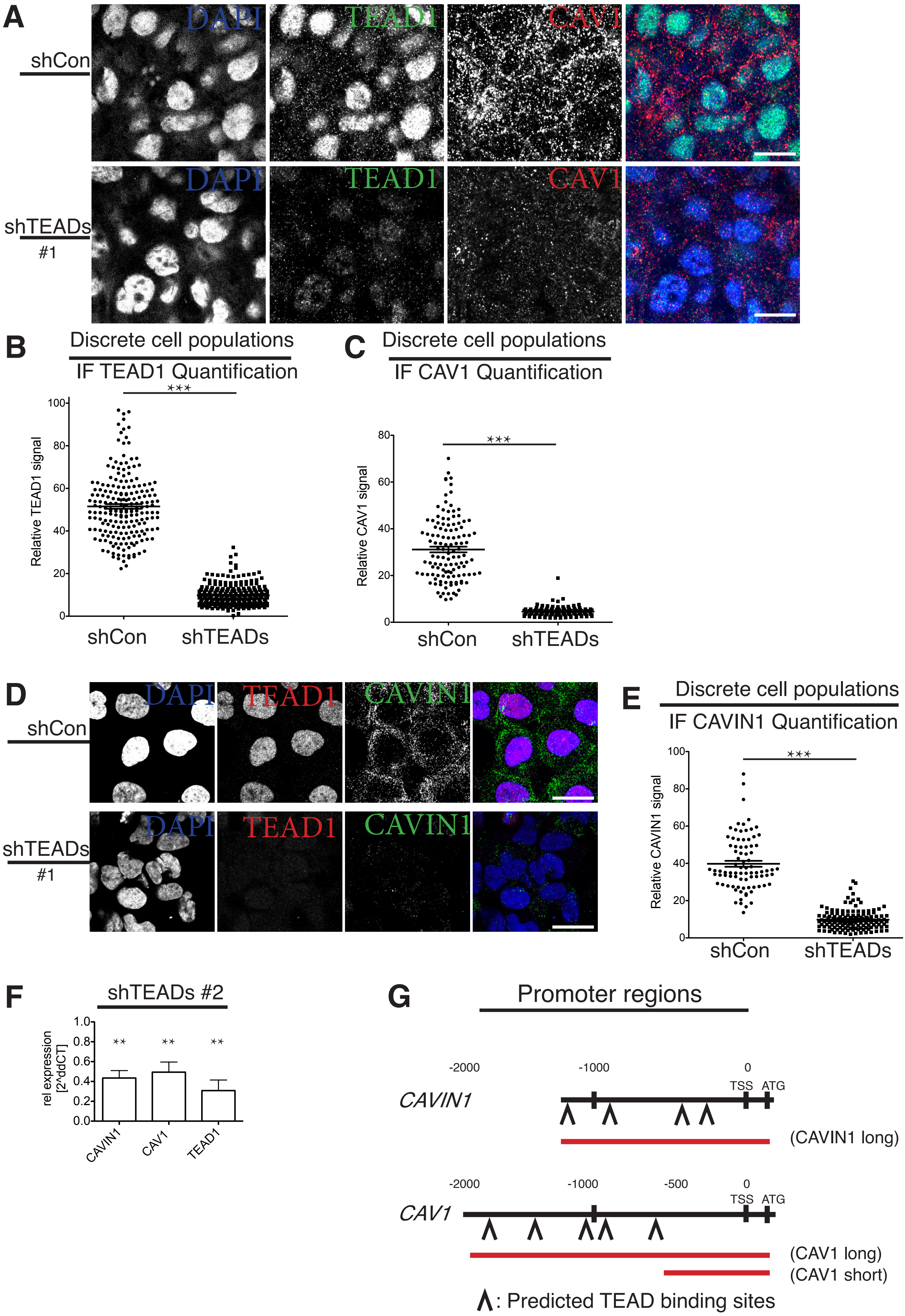
A
DAPI
TEAD1
CAV1
shCon
shTEADs
#1
B
Discrete cell populations
IF TEAD1 Quantification
Relative TEAD1 signal
***
100
80
60
40
20
0
shCon
shTEADs
C
Discrete cell populations
IF CAV1 Quantification
Relative CAV1 signal
***
80
60
40
20
0
shCon
shTEADs
D
DAPI
TEAD1
CAVIN1
shCon
shTEADs
#1
E
Discrete cell populations
IF CAVIN1 Quantification
Relative CAVIN1 signal
***
100
80
60
40
20
0
shCon
shTEADs
F
shTEADs #2
rel expression
[2^ddCT]
1.0
0.8
0.6
0.4
0.2
0.0
**
**
**
CAVIN1
CAV1
TEAD1
G
Promoter regions
-2000
-1000
0
TSS
ATG
CAVIN1
(CAVIN1 long)
-2000
-1000
-500
0
TSS
ATG
CAV1
(CAV1 long)
(CAV1 short)
: Predicted TEAD binding sites

Figure S4. TEADs regulate CAV1 and CAVIN1 expression. Related to Figure 3

(A) Discrete shCon (top) or shTEADs (bottom) HEK 293A cells were labeled for TEAD1 (red), CAV1 (green), and counterstained for DAPI (blue). Processing of samples for immunofluorescence and imaging was done in parallel with similar microscope settings to allow for direct comparison. Scale bars = 15μm**.** **(B)** Dot plot of TEAD1 levels from images, as shown in A. Each dot represents one cell. Means ± SEM. **(C)** Dot plot of CAV1 levels from images, as shown in A. Each dot represents one cell. Means ± SEM. **(D)** Discrete shCon (top) or shTEADs (bottom) HEK 293A cells were labeled for TEAD1 (red), CAVIN1 (green), and DAPI (blue). Scale bars = 30μm**.** Images are related to Figure 3C. **(E)** Dot plot of CAVIN1 levels from images, as shown in D. Each dot represents one cell. Means ± SEM. **(F)** qPCR of *CAV1* and *CAVIN1* mRNA levels in shTEADs #2 cells. For data on shTEADs #1 please see Figure 3F. **(G)** Diagram depicting the *CAVIN1* and *CAV1* proximal promoter regions, numbering is in base pairs (bp) from TSS. TSS: transcription start site. ATG: start codon. In red, representations of the regions contained in the luciferase reporter plasmids used in Figure 3G. Predicted TEAD (including degenerate) recognition motifs are depicted with arrow heads.

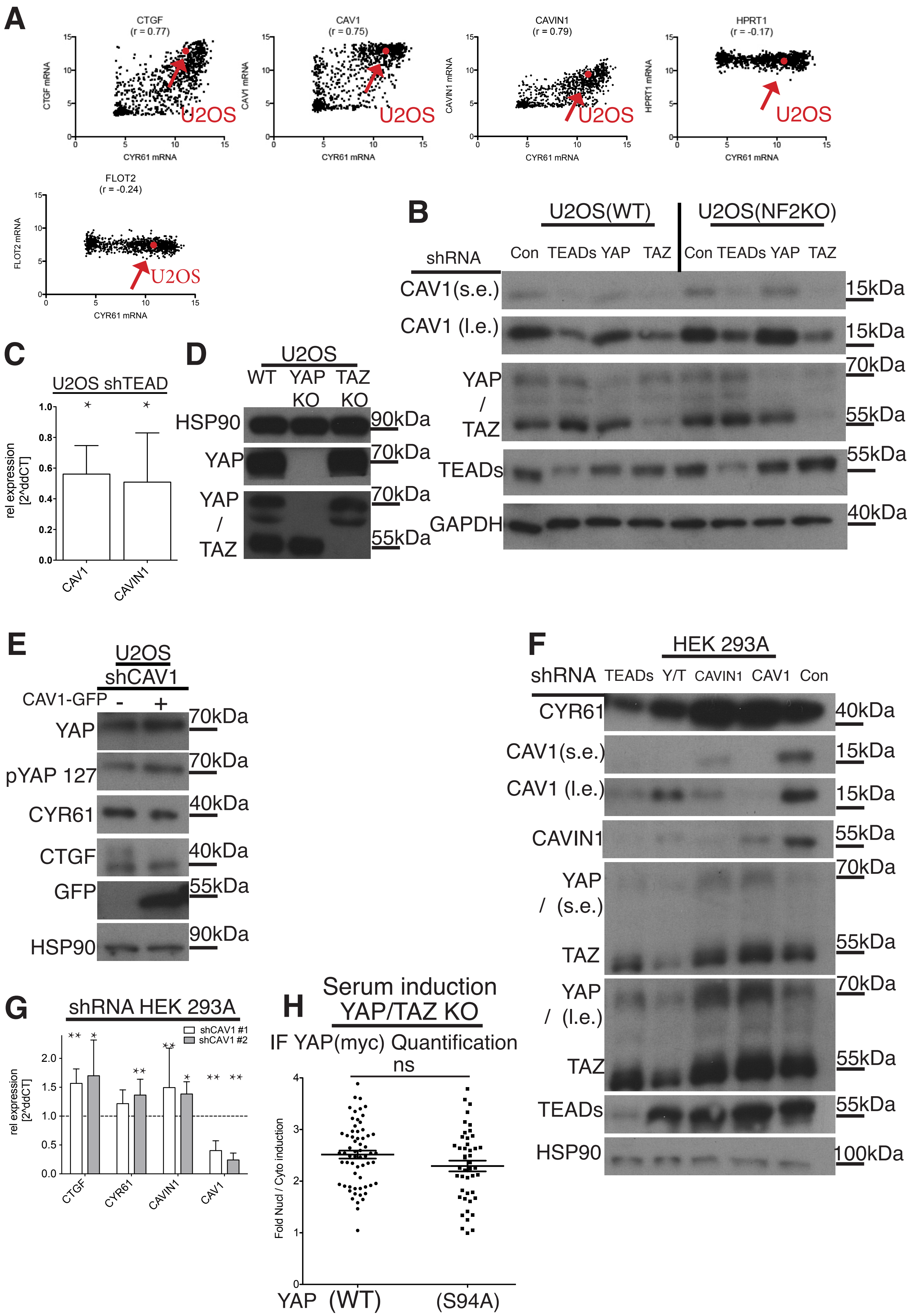
A
CTGF (r = 0.77)
CAV1 (r = 0.75)
CAVIN1 (r = 0.79)
HPRT1 (r = -0.17)
FLOT2 (r = -0.24)
CYR61 mRNA
U2OS
B
U2OS(WT)
U2OS(NF2KO)
shRNA
Con TEADs YAP TAZ Con TEADs YAP TAZ
CAV1(s.e.)
CAV1 (l.e.)
YAP / TAZ
TEADs
GAPDH
15kDa
70kDa
55kDa
40kDa
C
U2OS shTEAD
rel expression [2^ddCT]
CAV1
CAVIN1
D
U2OS
WT YAP KO TAZ KO
HSP90
YAP
YAP / TAZ
90kDa
E
U2OS shCAV1
CAV1-GFP - +
YAP
pYAP 127
CYR61
CTGF
GFP
HSP90
F
HEK 293A
shRNA TEADs Y/T CAVIN1 CAV1 Con
CYR61
CAV1(s.e.)
CAV1 (l.e.)
CAVIN1
YAP / (s.e.) TAZ
YAP / (l.e.) TAZ
TEADs
HSP90
100kDa
G
shRNA HEK 293A
shCAV1 #1
shCAV1 #2
CTGF
CYR61
CAVIN1
CAV1
H
Serum induction YAP/TAZ KO
IF YAP(myc) Quantification
ns
Fold Nucl / Cyto induction
YAP (WT)
(S94A)

Figure S5. CAV1 and CAVIN1 are YAP/TAZ-TEAD target genes across multiple cell types. Related to Figure 4,5 and 6

(A) Dot plots as shown in Figure 4A of *in silico* bioinformatics analysis of data obtained from CCLE. The localization of the U2OS cell line within the dot plot is highlighted with red circles. **(B)** Western blots from U2OS WT or NF2 KO cells with shRNA-induced knockdown of genes depicted. Short exposure (s.e.) and long exposure (l.e.). **(C)** Knockdown of *TEADs* caused reduced levels of *CAVIN1* and *CAV1* mRNA in U2OS WT cells, shown by qPCR analysis. Means ± SD. **(D)** Western blot of CrispR-mediated knockout of *YAP* or *TAZ* in U2OS cells used to generate cell lines analyzed in Figures 4B and C. **(E)** Western blots showing increased levels of pYAP 127 and decreased levels of CYR61 in CAV1 re-expressing U2OS cells. **(F)** Stable HEK 293A knockdown cell lines were processed for Western blotting. Short exposure (s.e), long exposure (l.e.). **(G)** mRNA levels of genes denoted were compared between shCon and two separate shCAV1 knockdown cell lines (#1 and #2) revealing YAP/TAZ hyperactivation in shCAV1 cells. Means ± SD. Cell lines in G were further analyzed in Figures 7F-H. **(H)** Scatterplot from immunofluorescence images from stable YAP/TAZ KO cell lines expressing either myc-tagged YAP, or YAP (S94A) labeled for YAP and DAPI. Cells were serum starved for two hours followed by serum feeding for two hours. Each dot represents one cell and is normalized to the average ratio of nuclear to cytoplasmic YAP in 2 hour serum starved cells processed in parallel and plotted as fold induction. The YAP-S94A mutant is regulated by Hippo pathway regulatory stimuli. N>50 for each condition. Not significant (ns), students T-test.

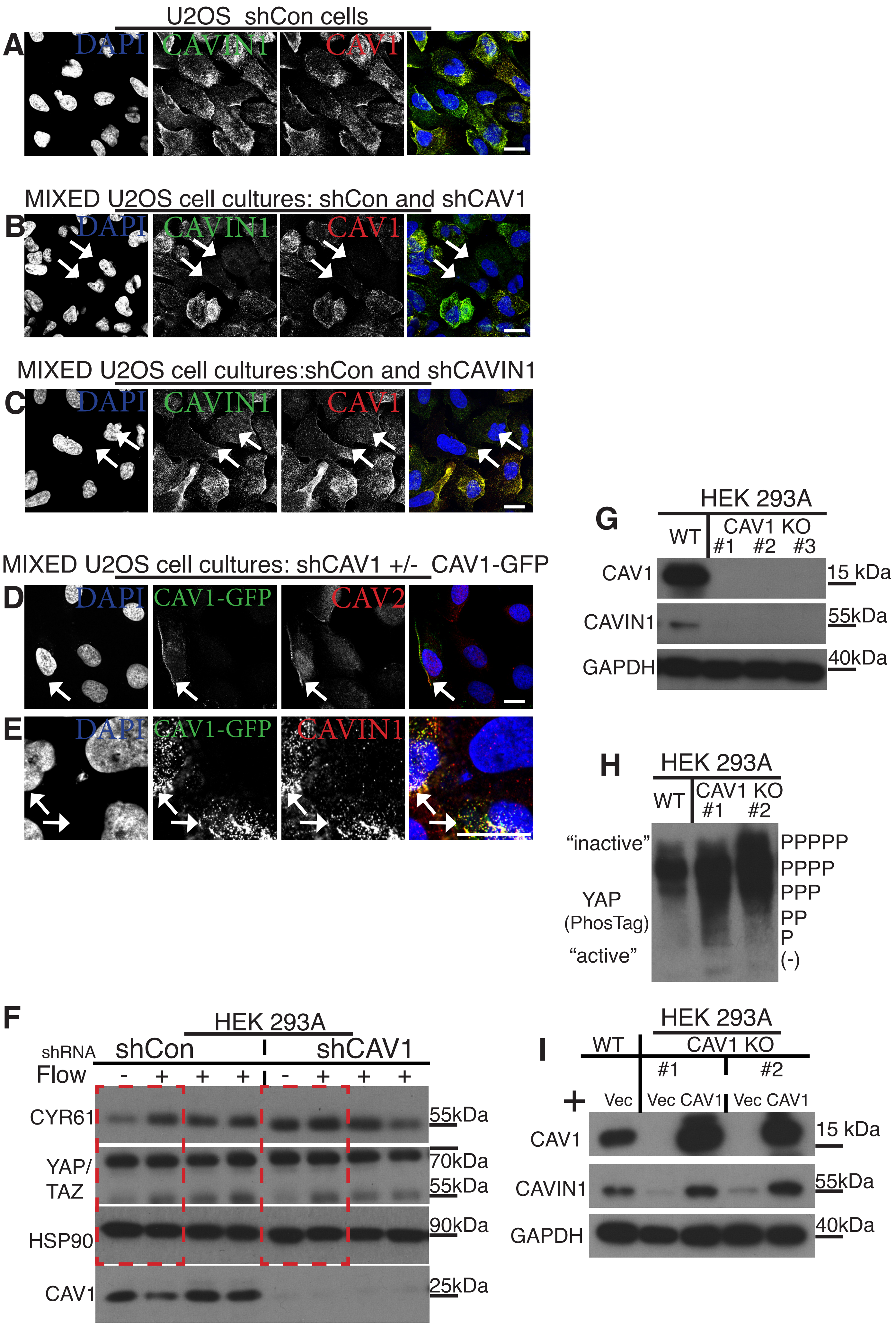

U2OS shCon cells
A
DAPI
CAVIN1
CAV1
MIXED U2OS cell cultures: shCon and shCAV1
B
DAPI
CAVIN1
CAV1
MIXED U2OS cell cultures:shCon and shCAVIN1
C
DAPI
CAVIN1
CAV1
MIXED U2OS cell cultures: shCAV1 +/- CAV1-GFP
D
DAPI
CAV1-GFP
CAV2
E
DAPI
CAV1-GFP
CAVIN1
F
HEK 293A
shRNA shCon shCAV1
Flow - + + + - + + +
CYR61
YAP/ TAZ
HSP90
CAV1
55kDa
70kDa
55kDa
90kDa
25kDa
G
HEK 293A
WT
CAV1 KO
#1 #2 #3
CAV1
CAVIN1
GAPDH
15 kDa
55kDa
40kDa
H
HEK 293A
WT
CAV1 KO
#1 #2
"inactive"
YAP (PhosTag)
"active"
PPPPP
PPPP
PPP
PP
P
(-)
I
HEK 293A
WT
CAV1 KO
#1 #2
+
Vec Vec CAV1 Vec CAV1
CAV1
CAVIN1
GAPDH
15 kDa
55kDa
40kDa

Figure S6. Specific CAV1 and CAVIN1 antibodies allow for single cell immunofluorescence based studies. Related to Figure 5 and 7

(A) Confocal image of shCAV1 U2OS cells labeled for CAVIN1 (green), CAV1 (red) and counterstained with DAPI (blue). **(B)** Confocal image of mixed cell culture of shCAV1 and shCon U2OS cells. Cells were labeled for CAVIN1 (green), CAV1 (red), and DAPI (blue). Arrows: Examples of shCAV1 cells. Note shCAV1 cells have decreased levels of CAVIN1 signal. Figures S6A, B together with Figures S7C-E validate the use of the CAV1 antibody in mixed cell assays as those shown in Figures 5B and 7I,J. **(C)** Mixed cell culture of shCAVIN1 and shCon U2OS cells. Cells labeled for CAVIN1 (green), CAV1 (red), and DAPI. Arrows: Examples of shCAVIN1 cells. Note shCAVIN1 cells have decreased levels of CAV1 signal. Scale bars A-C = 20μm. **(D)** Confocal image of mixed cell populations. A shCAV1 U2OS cell re-expressing CAV1 (CAV1-GFP) is highlighted with an arrow. The cells were labeled for GFP (green), CAV2 (red) and DAPI (blue). Scale bar = 10μm. **(E)** Confocal image of mixed cell populations as in D. Cells were labeled for GFP (green), CAVIN1 (red) and DAPI (blue). Scale bar = 20μm. Figure S6D and Figure S6E illustrate rescue of functional CAV1 upon exogenous CAV1-GFP expression, as CAV2 and CAVIN1 localize to CAV1 positive plasma membrane domains. **(F)** Western blot of HEK 293A cell lysates from either control or shCAV1#2 cells were kept at either 0 (- flow) or at $2.1*10^{-5}$ Dyn/cm^2 (+ flow). Red box indicates regions shown in Figure 7F. **(G)** Western blots of HEK 293A WT and CrispR-mediated CAV1KO cell lines generated. #2 was generated using a separate guide sequence compared to #1 and #3. **(H)** PhosTag gel-based Western blot probed against YAP from cell lysates of HEK 293A WT and CAV1 KO#1 and #2. **(I)** Western blots of WT cells compared to CAV1 KO#1 and #2 vector control cells as well as CAV1 KO#1 and #2 cells re-expressing CAV1.

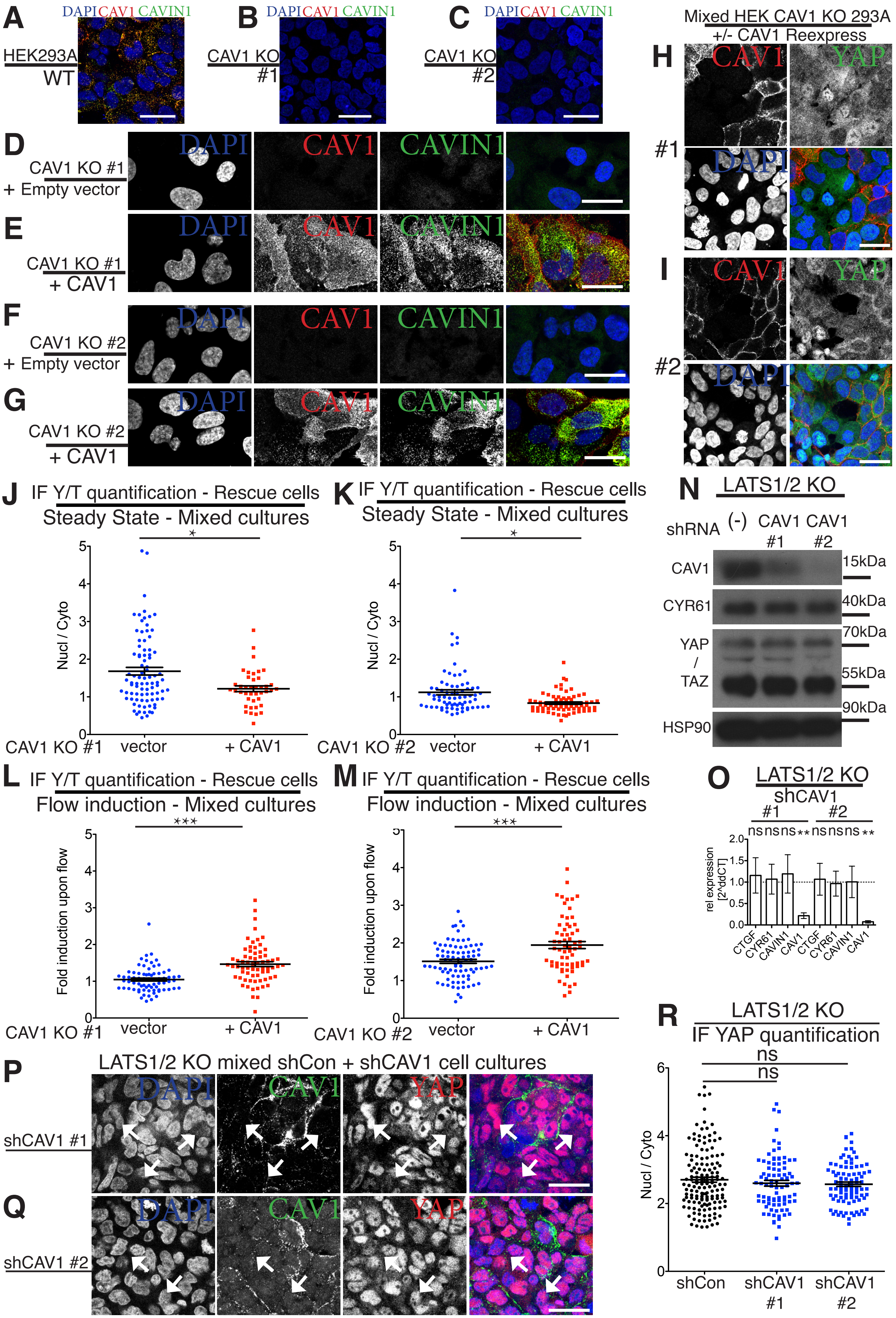
A
DAPI CAV1 CAVIN1
HEK293A WT
B
DAPI CAV1 CAVIN1
CAV1 KO #1
C
DAPI CAV1 CAVIN1
CAV1 KO #2
D
CAV1 KO #1 + Empty vector
E
CAV1 KO #1 + CAV1
F
CAV1 KO #2 + Empty vector
G
CAV1 KO #2 + CAV1
DAPI
CAV1
CAVIN1
Mixed HEK CAV1 KO 293A +/- CAV1 Reexpress
H
I
#1
#2
CAV1
YAP
DAPI
J
IF Y/T quantification - Rescue cells
Steady State - Mixed cultures
Nucl / Cyto
CAV1 KO #1
vector
+ CAV1
K
IF Y/T quantification - Rescue cells
Steady State - Mixed cultures
CAV1 KO #2
N
LATS1/2 KO
shRNA
(-)
CAV1 #1
CAV1 #2
CAV1
CYR61
YAP / TAZ
HSP90
15kDa
40kDa
70kDa
55kDa
90kDa
L
IF Y/T quantification - Rescue cells
Flow induction - Mixed cultures
Fold induction upon flow
M
IF Y/T quantification - Rescue cells
Flow induction - Mixed cultures
O
LATS1/2 KO
shCAV1
#1
#2
ns ns ns ** ns ns ns **
rel expression [2^ddCT]
CTGF
CYR61
CAVIN1
CAV1
R
LATS1/2 KO
IF YAP quantification
ns
ns
shCon
shCAV1 #1
shCAV1 #2
P
LATS1/2 KO mixed shCon + shCAV1 cell cultures
shCAV1 #1
Q
shCAV1 #2
DAPI
CAV1
YAP

Figure S7. CAV1 rescue cells functionally rescue the observed CAV1-deficient phenotype. Related to Figures 2 and 7.
(A) Confocal image of HEK 293A WT labeled for CAV1 (red), CAVIN1 (green), and counterstained for DAPI (blue). **(B)** HEK 293A CAV1 KO#1 cells and **(C)** HEK 293A CAV1 KO#2 cells labeled and imaged as cells in C. Scale bars A-C = 30µm. **(D)** Confocal image of HEK 293A CAV1 KO#1 expressing empty vector and **(E)** re-expressing CAV1 and labeled for CAV1 (red), CAVIN1 (green), and DAPI (blue). **(F)** Confocal image of HEK 293A CAV1 KO#2 expressing empty vector and **(G)** re-expressing CAV1 and labeled for CAV1 (red), CAVIN1 (green) and DAPI (blue). Scale bars D-G = 30µm. **(H)** Confocal images of mixed cell cultures of CAV1 KO#1 and **(I)** CAV1 KO#2 expressing empty vector or re-expressing CAV1 labeled for CAV1 (red), YAP (green), and DAPI (blue). Scale bars H,I= 25µm. **(J)** Dot plot of quantification of YAP localization from confocal images of mixed HEK 293A cell cultures. Each dot represents one cell. The cells were labeled for CAV1, YAP, and DAPI, which allowed for determination of nuclear to cytoplasmic (Nucl/Cyto) localization of YAP in CAV1 KO#1 vector control (blue) or CAV1 re-expressing cells (red). Cells were cultured at steady state. **(K)** Quantification as in J but of CAV1 KO#2 cells. **(L)** Cells as in J but of CAV1 KO#1 cultured at $2.1*10^{-5}$ Dyn/cm^2 and processed in parallel with samples in J. The nuclear to cytoplasmic ratio was normalized to average of steady state conditions. **(M)** Quantification as in L but of CAV1 KO#2. C-F: Means ± SEM. **(N)** Western blot analysis of lysates from LATS1/2 KO with and without shCAV1-induced knockdown. **(O)** qPCR analysis of cell lines as in G and compared to LATS1/2 KO cells. Means ± SD. **(P)** Confocal image of mixed cells cultures of LATS1/2 with and without shCAV1#1 or **(Q)** shCAV1#2-induced *CAV1* knockdown. Labeled against CAV1 (green), YAP (red), and DAPI (blue). Arrows highlight examples of *CAV1* knockdown cells. Scale bars P,Q = 25µm. **(R)** Quantification of YAP nuclear to cytoplasmic localization from images as those in P and Q. Each dot represents one cell. Means ± SEM. No significant difference (ns) was observed between groups.

Table S1: Sequence of primers used for qRT-PCR (5'→3'). Related to STAR Methods

Target	Forward Primer	Reverse Primer
CAV1	GCGACCCTAAACACCTCAAC	ATGCCGTCAAAACTGTGTGTC
CAV1 prom	TGGCATAACCTGTTGGCATA	CCCAAACGCTTCGAAATAAG
CAV1 ingene	CCTCCGTGTCTCAGTGGTTT	TCACCTTGCTTGCCTTTCTT
cav1 fish	CGATGTGGTGAAGGTGGACTTT	TCAGCAGCCTGTAGCACCAAT
CAVIN1	GAGGACCCCACGCTCTATATT	CCCCGATGATTTTGTCCAGGA
CAVIN1 prom	TTTCAGAATCTCCTGGTCCAC	TTGCTCTCTGTTTCCCTCTCA
CAVIN1 ingene	GCAGTTTTGAGGAGGCAAAG	AAATGCTTCCTGGCCCTTAT
cavin1b fish	AAACGTCTGGAGAGCAACGAGA	GCCACA TTCACTTTCGAACCC
CTGF	CCAATGACAACGCCTCCTG	TGGTGCAGCCAGAAAGCTC
CTGF prom	CTTTGGAGAGTTTCAAGAGCC	TCTGTCCACTGACATACATCC
ctgf fish	CTGCACAGCCAGAGATG	CACTTCCCAGGCACTTT
CYR61	AGCCTCGCATCCTATACAACC	TTCTTTCACAAGGCGGCACTC
cyr61 fish	CCGTGTCCACATGTACATGGG	GGTGCATGAAAGAAGCTCGTC
ef1a fish	TCTCTCAATCTTGAAACTTATCAATCA	AACACCCAGGCGTACTTGAA
TEAD1	ATGGAAAGGATGAGTGACTCTGC	TCCCACATGGTGGATAGATAGC
YAP	CCAAGGCTTGACCCTCGTTTTG	TCGCATCTGTTGCTGCTGGTTG
TAZ (WWTR1)	AATGGAGGGCCATATCATTCGAG	GTCCTGCGTTTTCTCCTGTATC
HPRT1	AGAATGTCTTGATTGTGGAAGA	ACCTTGACCATCTTTGGATTA

If not stated otherwise, primers are specific to human genes. All primers have been obtained from Integrated DNA Technologies, Inc.

Table S2: Guide sequences utilized for CrispR/Cas9-mediated knockout (5'→3'). Related to STAR Methods

Target	Forward Primer	Reverse Primer
CAV1#1	CACCGAGTGTACGACGCGCACACCA	AAACTGGTGTGCGCGTCGTACACTC
CAV1#2	CACCGTTTAGGGTCGCGGTTGACC	AAACGGTCAACCGCGACCCTAAAC
YAP#1	CACCGCATCAGATCGTGCACGTCCG	AAACCGGACGTGCACGATCTGATGC
WWTR1 (TAZ)#1	CACCGTGTCTAGGTCCTGCGTGACG	AAACCGTCACGCAGGACCTAGACAC
NF2	CACCGTCCATGGTGACGATCCTCA	AAACTGAGGATCGTCACCATGGAC

Table S3: Plasmids used. Related to STAR Methods

Target	Source	Identifier
pMD2.G	Professor Kun-Liang Guan lab, University of California, San Diego (UCSD)	N/A
pSPAX2	Professor Kun-Liang Guan lab, University of California, San Diego (UCSD)	N/A
pLKO.1	Professor Kun-Liang Guan lab, University of California, San Diego (UCSD)	N/A
Cav1-GFP	Originally from Ari Helenius Addgene #14433.	N/A
pBabe-CAV1	This study (generated by GeneWIZ)	N/A
pBabe	Addgene	#1764
pSpCas9(BB)-2A-Puro (PX459)	Addgene	#48139
pSpCas9(BB)-2A-Puro (PX459) V2.0	Addgene	#48139
pCMV Flag-YAP	Professor Kun-Liang Guan lab, University of California, San Diego (UCSD)	N/A
pQCXIH-Myc-YAP (S5A)	Professor Kun-Liang Guan lab, (UCSD) (Note also encodes S128A, S131A, and S163A) Addgene	#48139
pGL3-basic	Promega	E1751
pGL3 CAV1 long	This study	N/A
pGL3 CAV1 short	This study	N/A
pGL3 CAVIN1 long	This study	N/A
pcDNA3	Ben Nichols, MRC LMB, Cambridge UK	N/A
pQCXIH-Myc YAP	Addgene	#33091
pQCXIH-Myc YAP S94A	Addgene	#33094
pBabe-Flag TAZ (WT)	Professor Kun-Liang Guan lab, University of California, San Diego (UCSD)	N/A
pBabe-Flag TAZ (S89A)	Professor Kun-Liang Guan lab, University of California, San Diego (UCSD)	N/A
pQCXIH-HA LATS1 (WT)	Professor Kun-Liang Guan lab, University of California, San Diego (UCSD)	N/A

Table S3: Plasmids used (continued)

pQCXIH-HA LATS1 (K/R)	Professor Kun-Liang Guan lab, University of California, San Diego (UCSD)	N/A
tol2 - *4.4kb*krt18:eGFP-YapS54A	Link lab, Medical College of Wisconsin	This study
tol2 h2ax:H2A-mCherry	Link lab, Medical College of Wisconsin	This study
shRNA CAV1 #1	Sigma-Aldrich	TRCN0000007999
shRNA CAV1 #2	Sigma-Aldrich	TRCN0000008002
shRNA CAVIN1 #1	Sigma-Aldrich	TRCN0000430242
shRNA CAVIN1 #2	Sigma-Aldrich	TRCN0000446514
shRNA YAP	Sigma-Aldrich	TRCN0000300325
shRNA TAZ	Sigma-Aldrich	TRCN0000370007
shRNA TEAD1/3/4 #1 and #2	Professor Kun-Liang Guan lab, University of California, San Diego (UCSD)	Zhao et al., 2008 [S1]

All shRNA constructs carry the pLKO.1 backbone (puromycin selection)

Supplemental Reference List

[S1] Zhao, B., Ye, X., Yu, J., Li, L., Li, W., Li, S., Yu, J., Lin, J.D., Wang, C.Y., Chinnaiyan, A.M., et al. (2008). TEAD mediates YAP-dependent gene induction and growth control. Genes & development *22*, 1962-1971.
