# Supplementary material for: The Hippo Pathway Regulates Caveolae Expression and Mediates Flow Response via Caveolae
Source: Curr Biol. 2019 Jan 21;29(2):242–255.e6. doi: 10.1016/j.cub.2018.11.066 (PMC6345631; doi:10.1016/j.cub.2018.11.066)

Full scans of WBs shown in Figure 1F

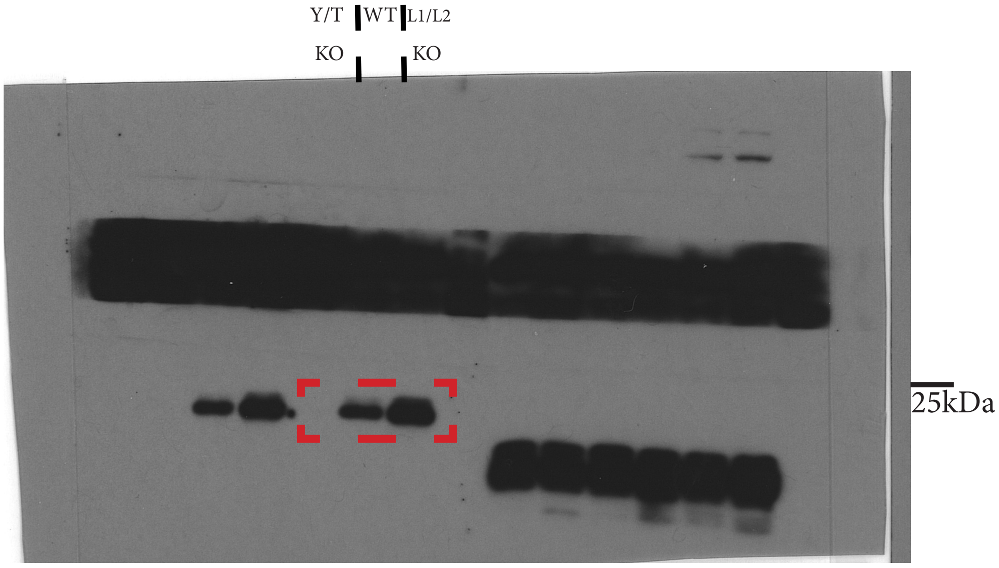

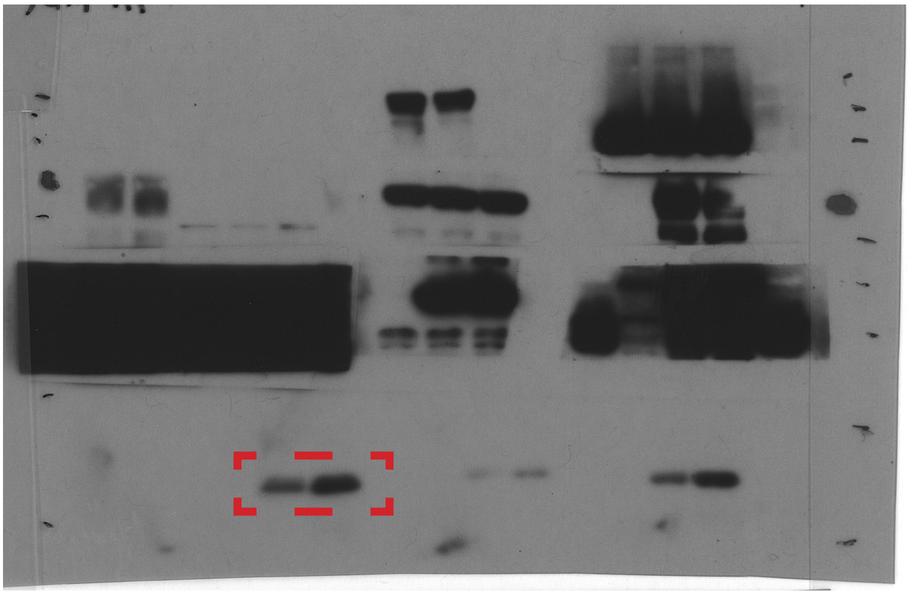

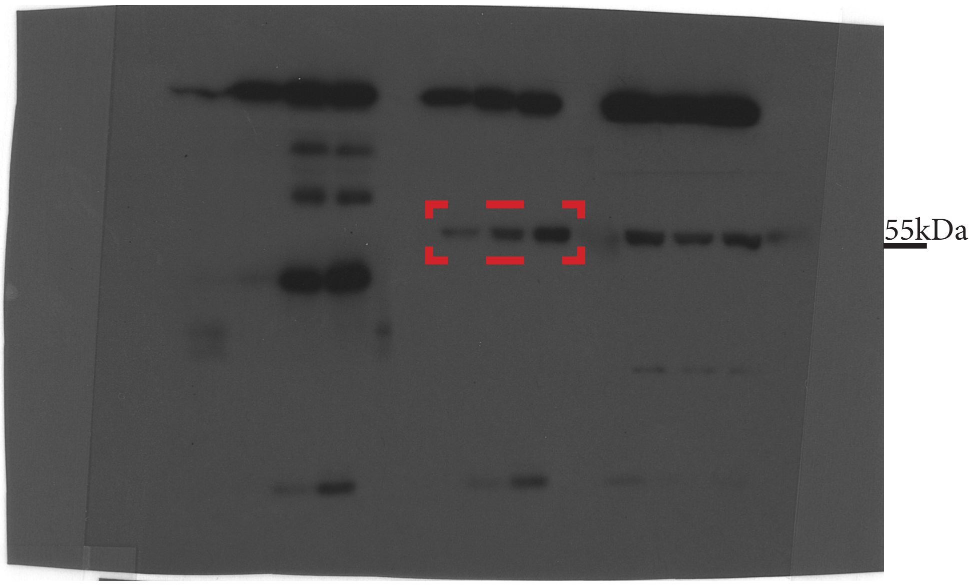

Full scans of WBs shown in Figure 1F (continued)

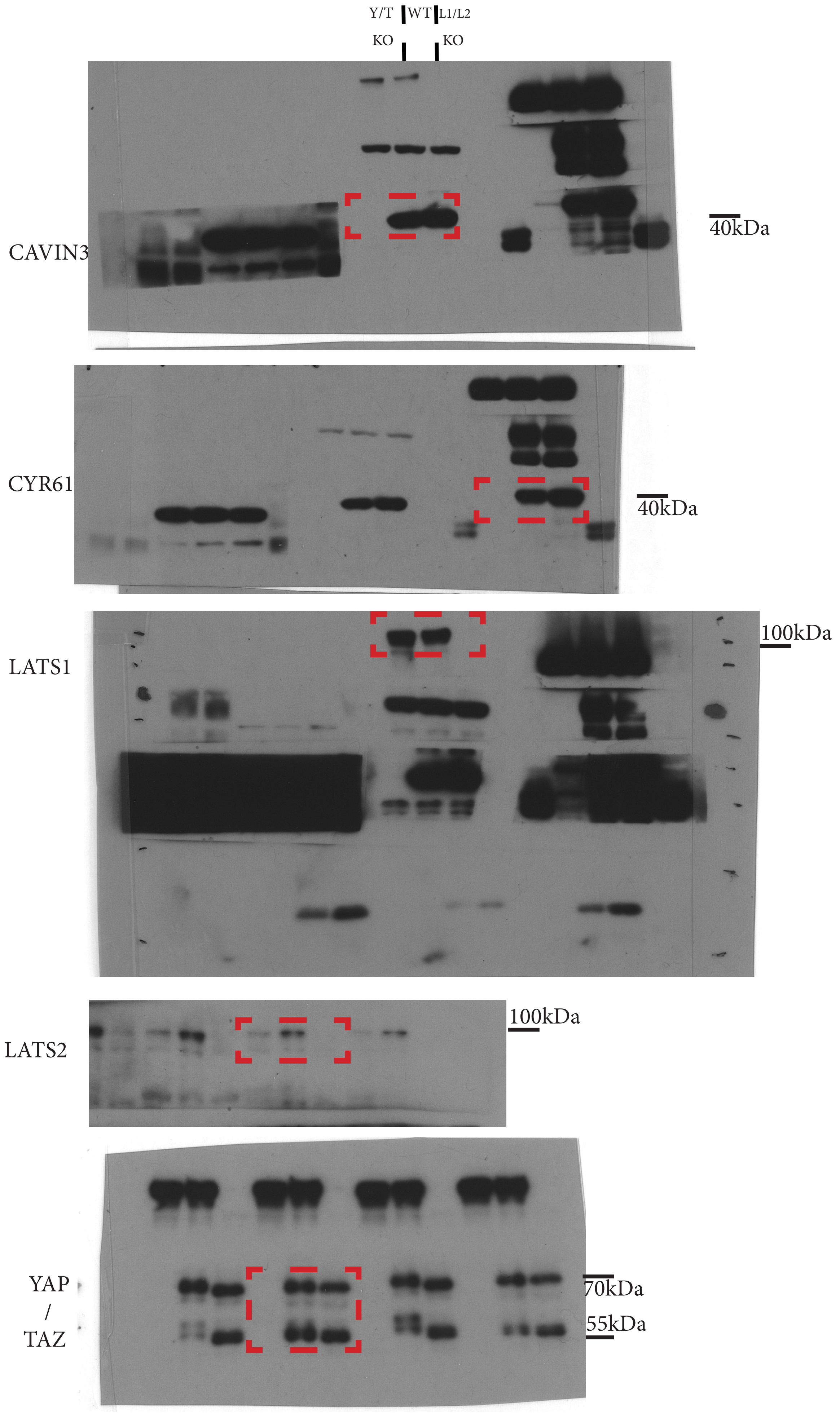

Full scans of WBs shown in Figure 1F (continued)

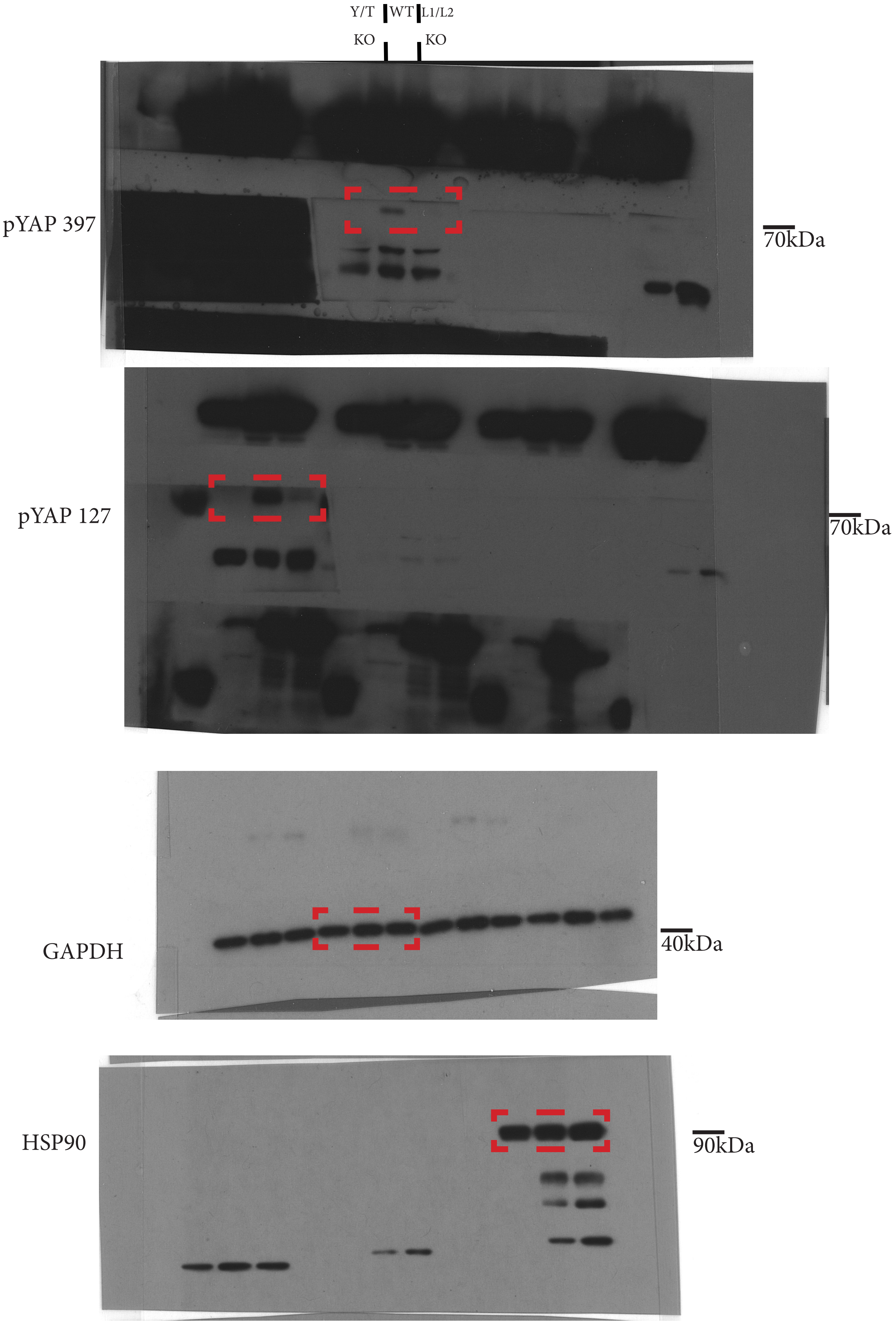

Full scans of WBs shown in Figure 1G

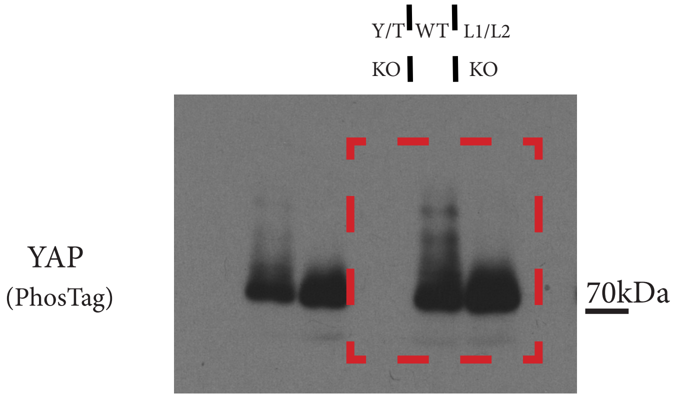

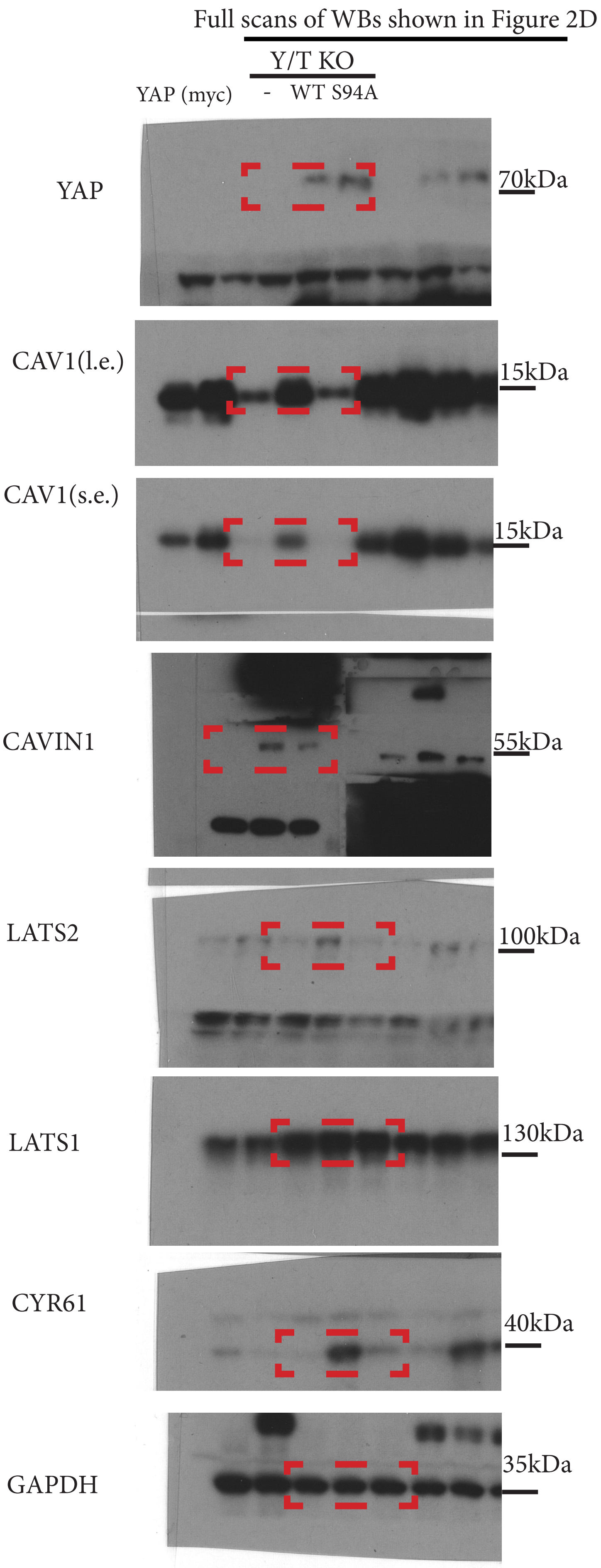
Full scans of WBs shown in Figure 2D
Y/T KO
YAP (myc)
-
WT
S94A
YAP
70kDa
CAV1(l.e.)
15kDa
CAV1(s.e.)
15kDa
CAVIN1
55kDa
LATS2
100kDa
LATS1
130kDa
CYR61
40kDa
GAPDH
35kDa

Full scans of WBs shown in Figure 3A

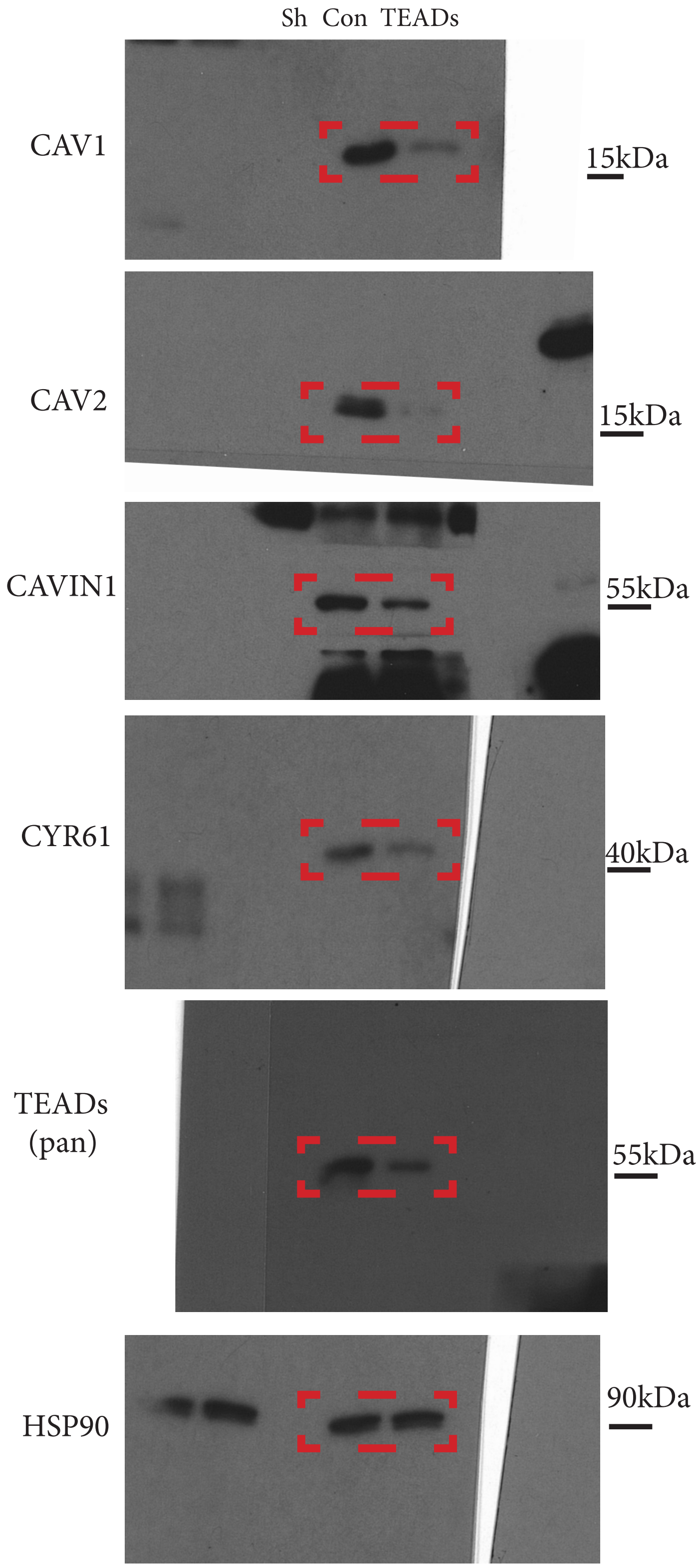

Full scans of WBs shown in Figure 4B

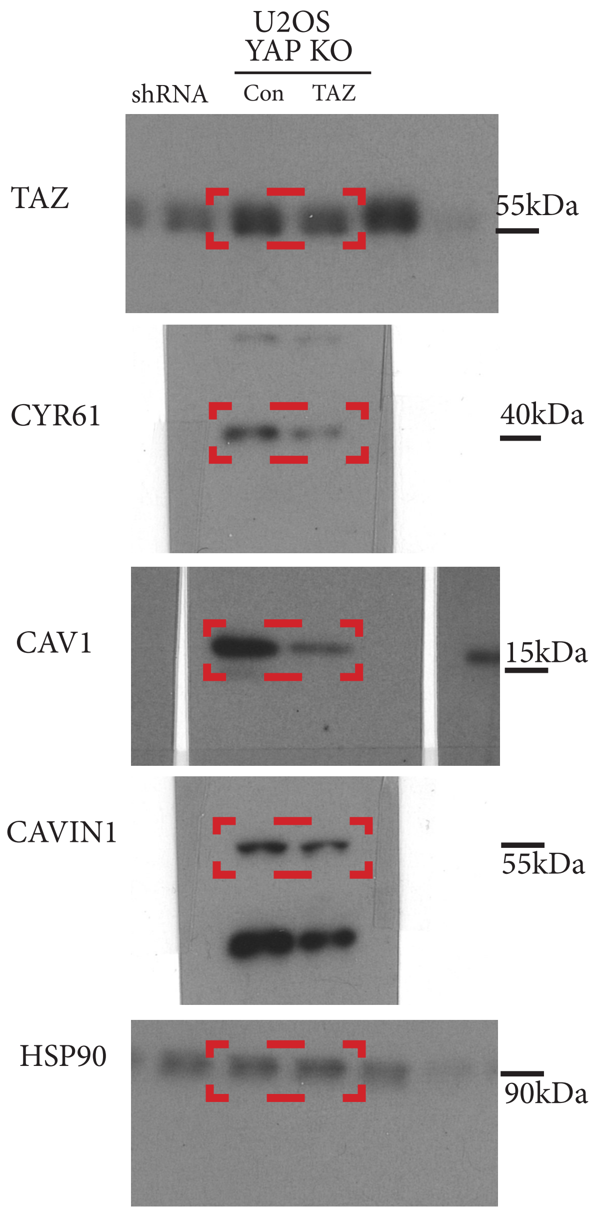

Full scans of WBs shown in Figure 4C

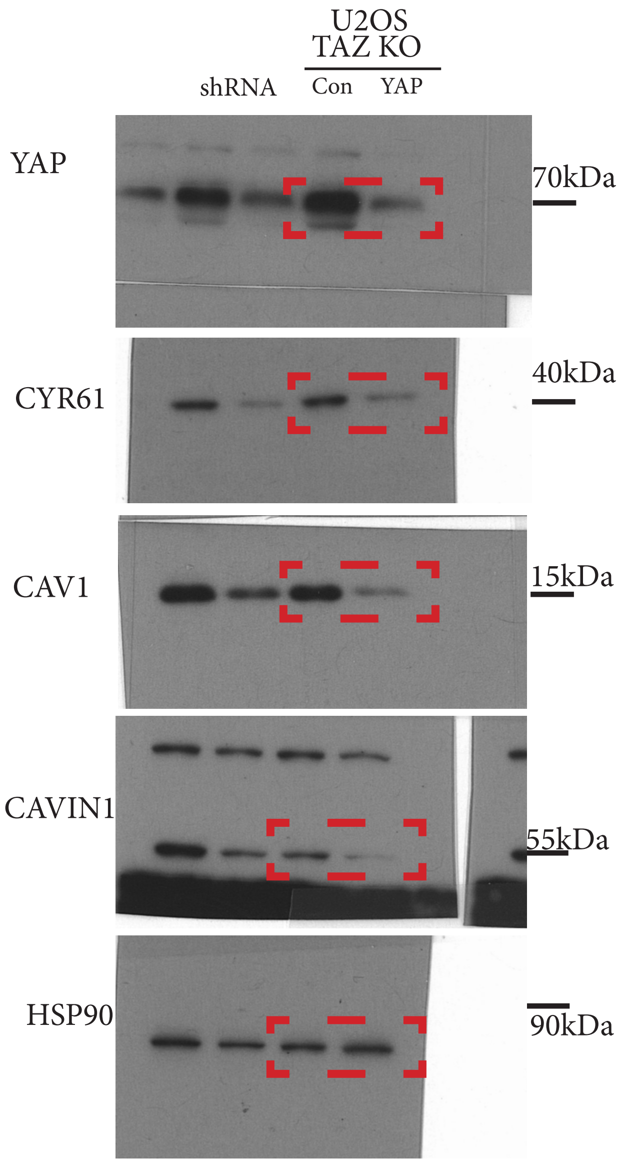

Full scans of WBs shown in Figure 4D

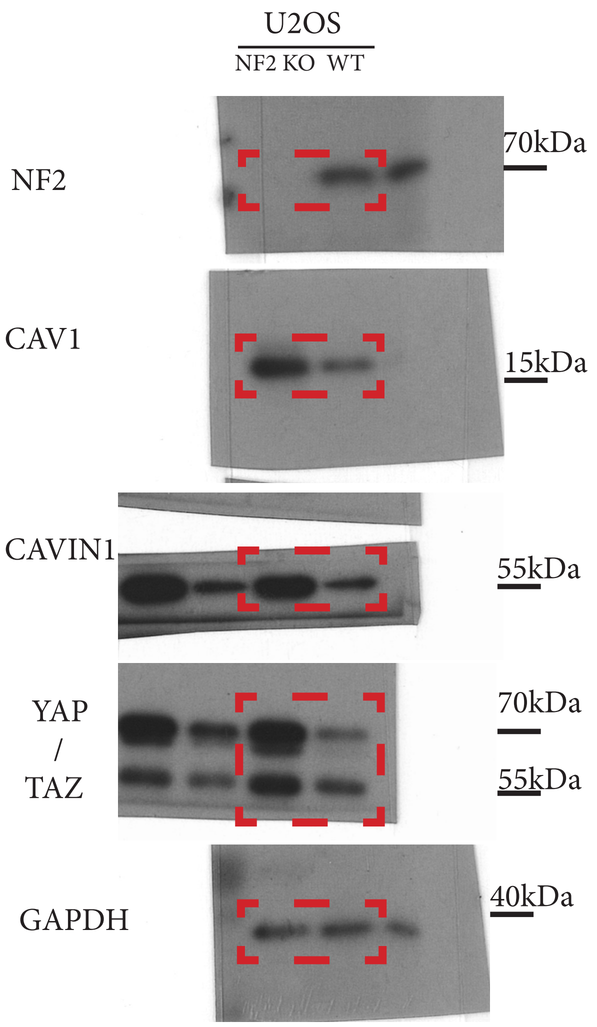

Full scans of WBs shown in Figure 4H

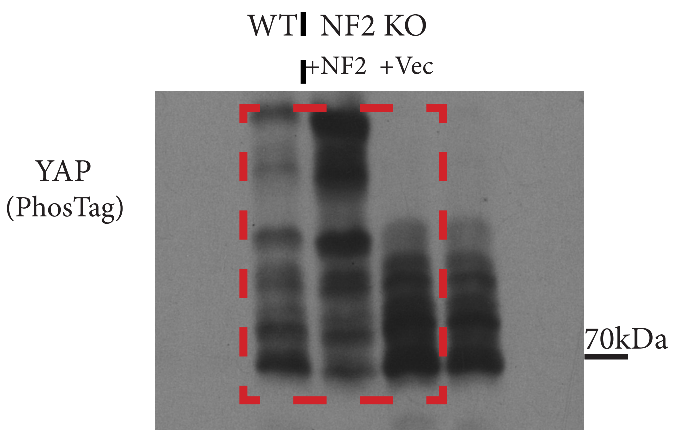

Full scans of WBs shown in Figure 4I

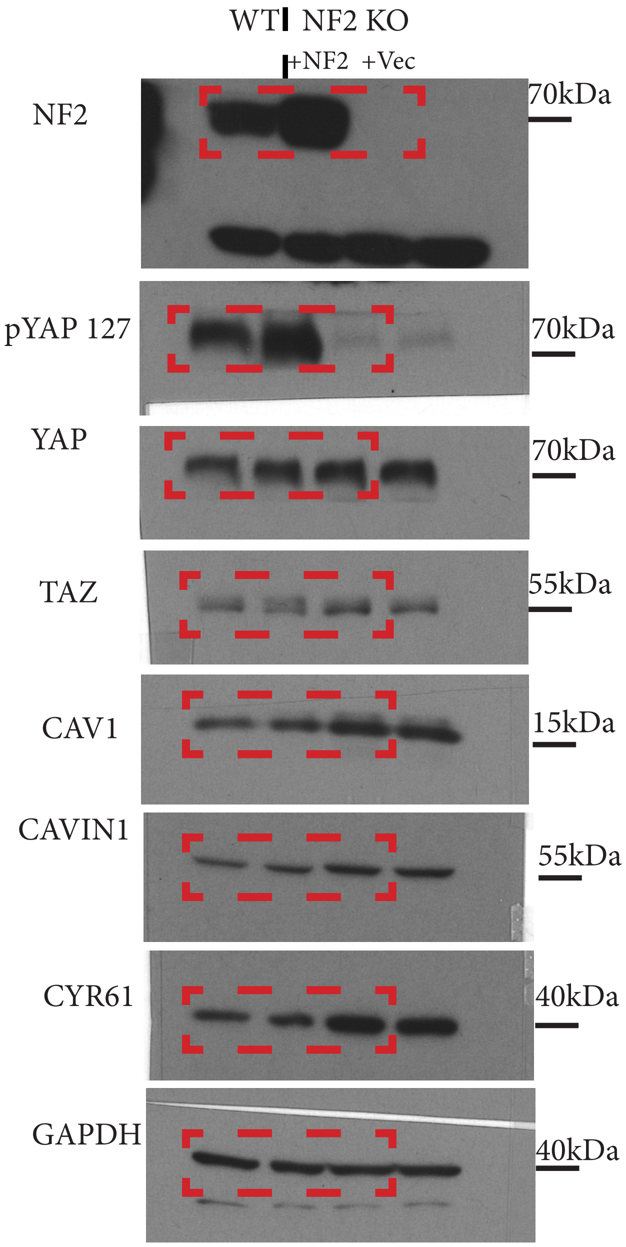

Full scans of WBs shown in Figure 5A

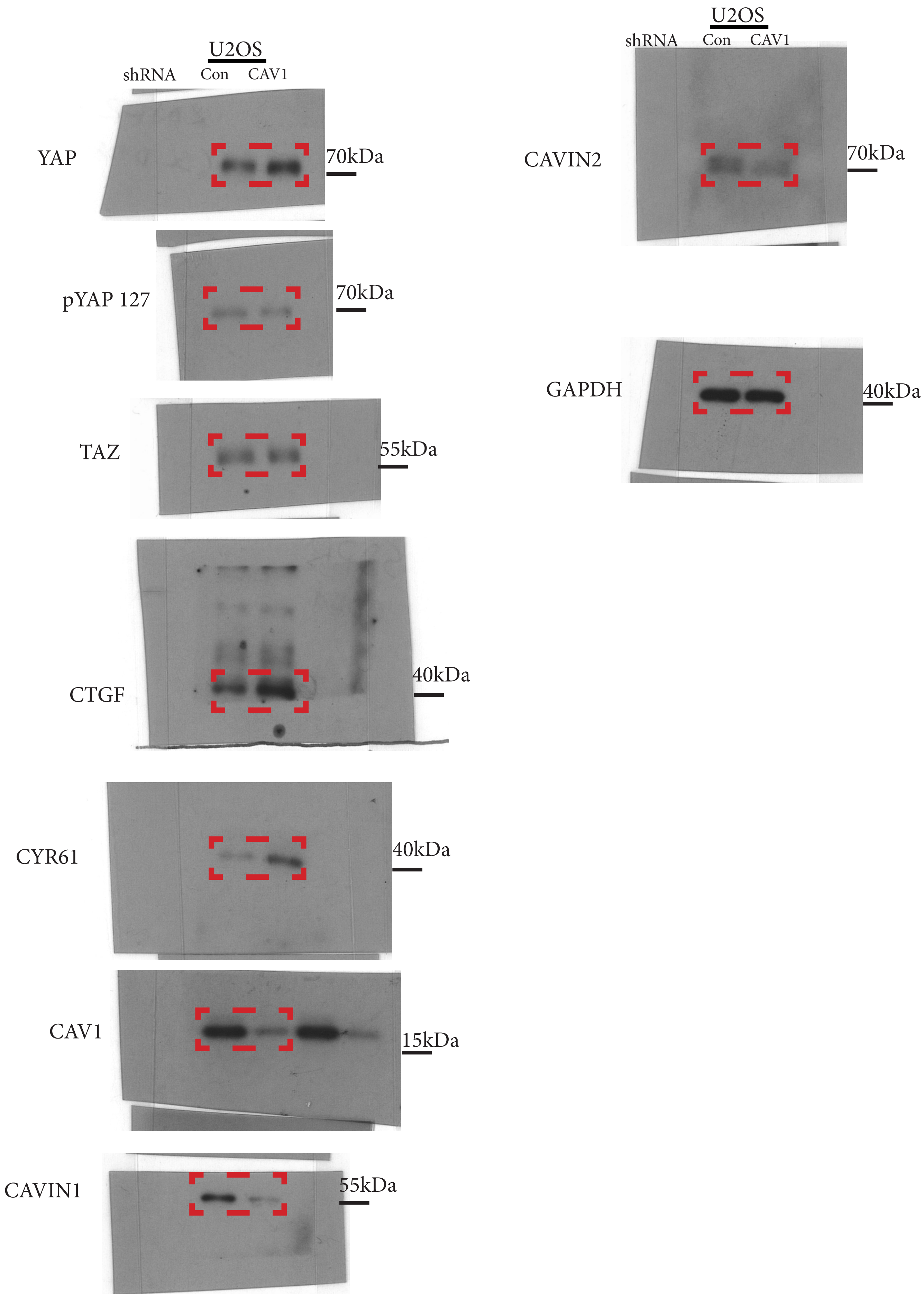

Full scans of WBs shown in Figure 5E

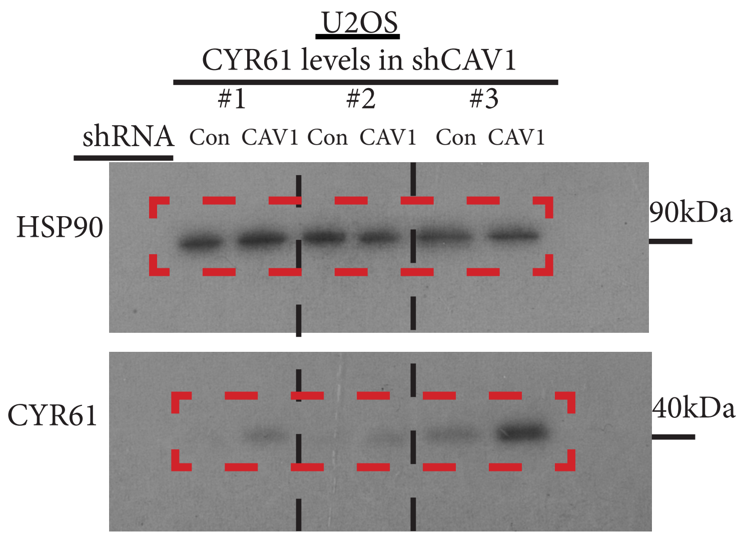

Full scans of WBs shown in Figure 7A

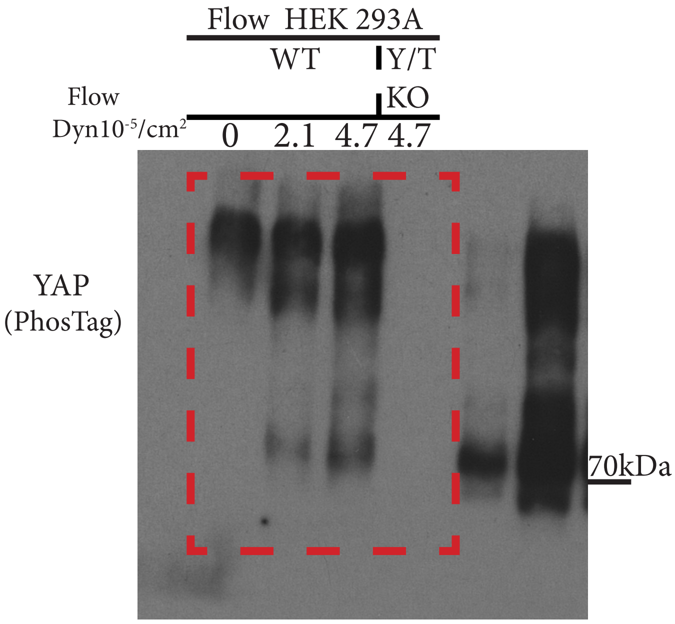

Full scans of WBs shown in Figure 7B

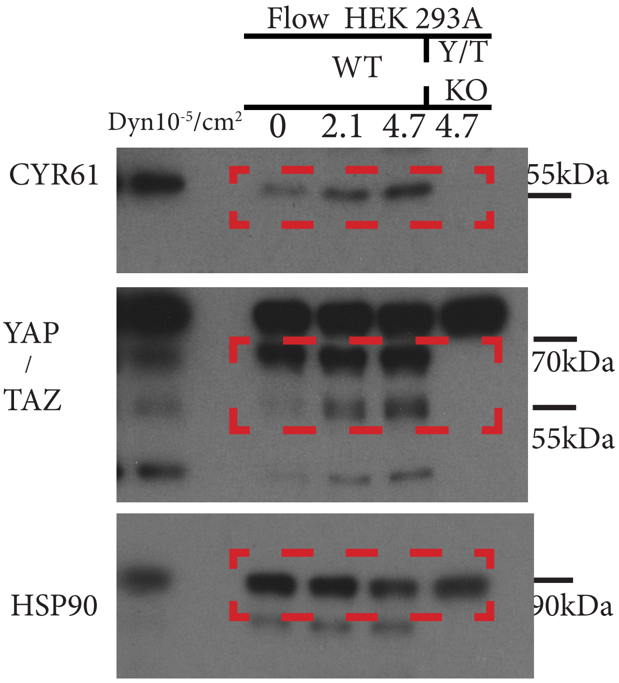

Full scans of WBs shown in Figures 7F and S6F

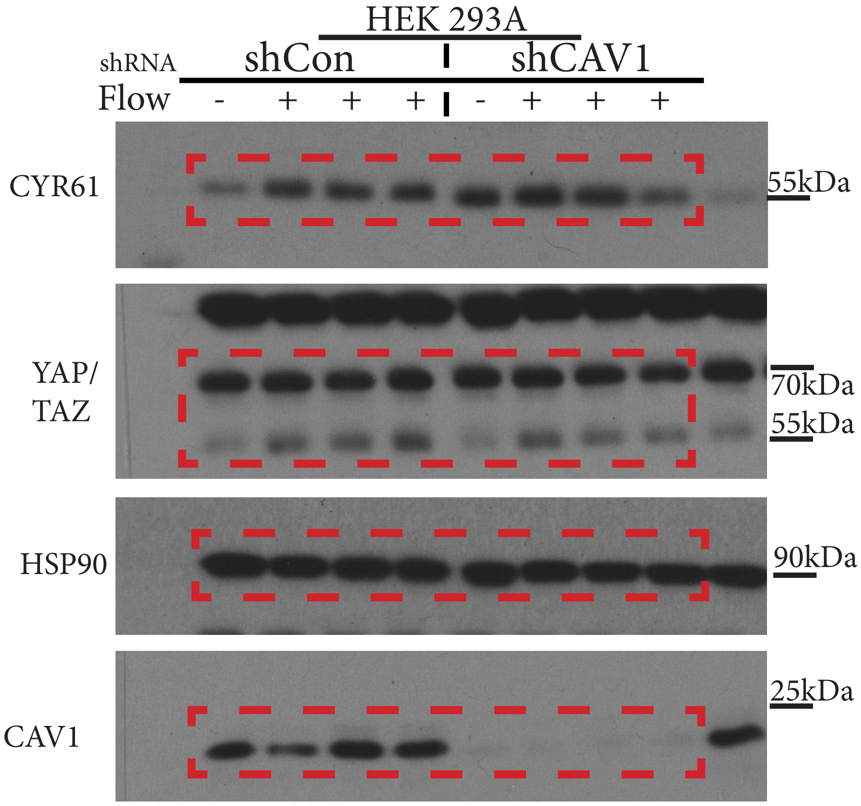

Full scans of WBs shown in Figure S2E

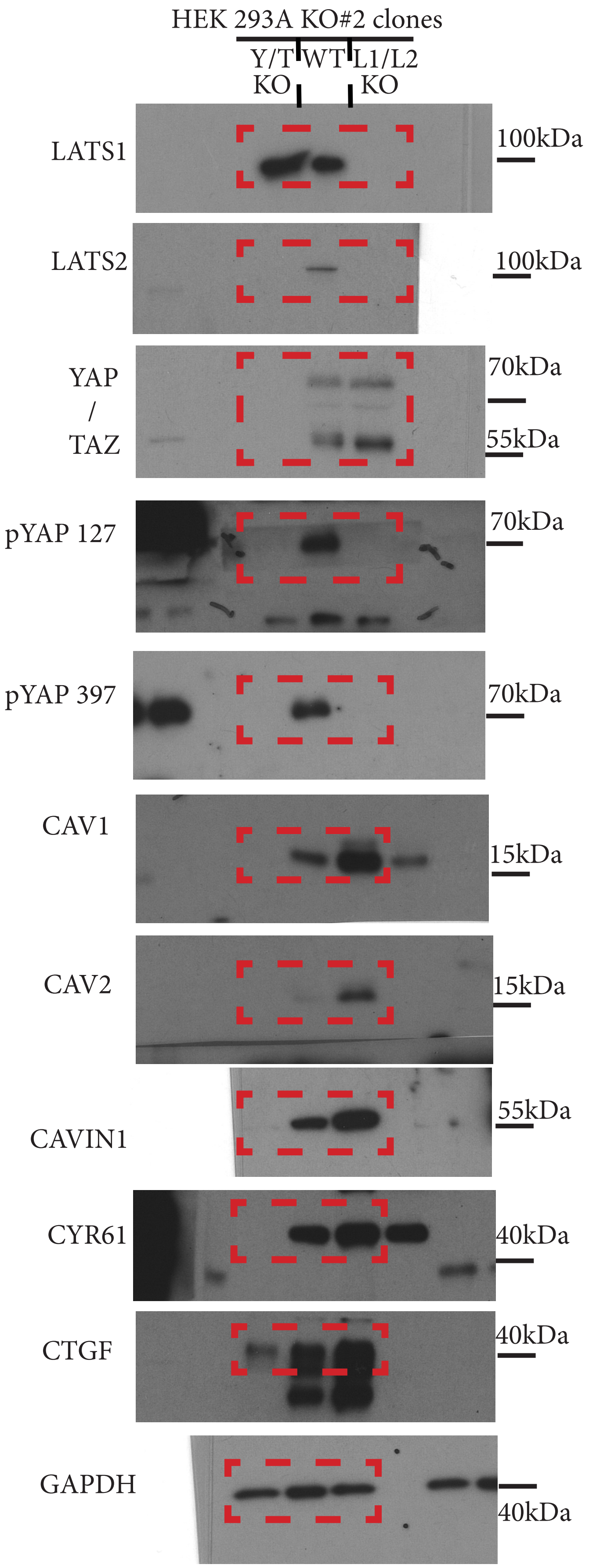

Full scans of WBs shown in Figure S2G

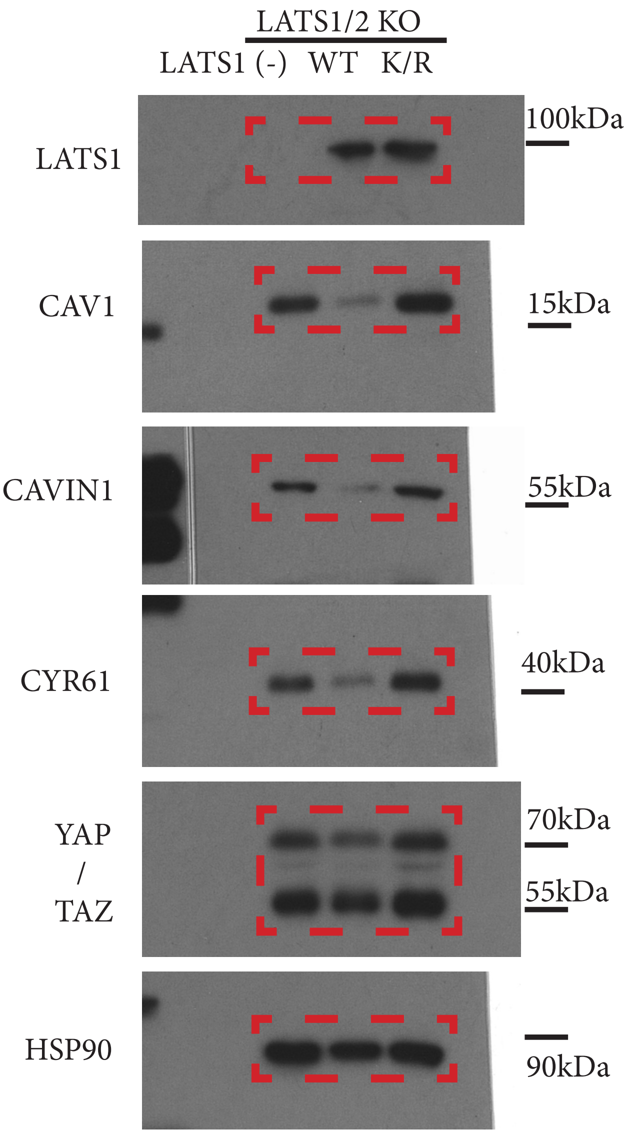

Full scans of WBs shown in Figure S2H

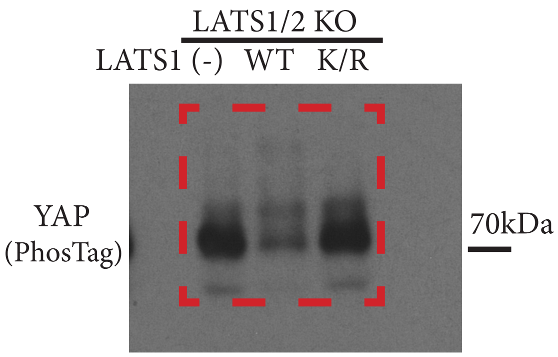

Full scans of WBs shown in Figure S5B

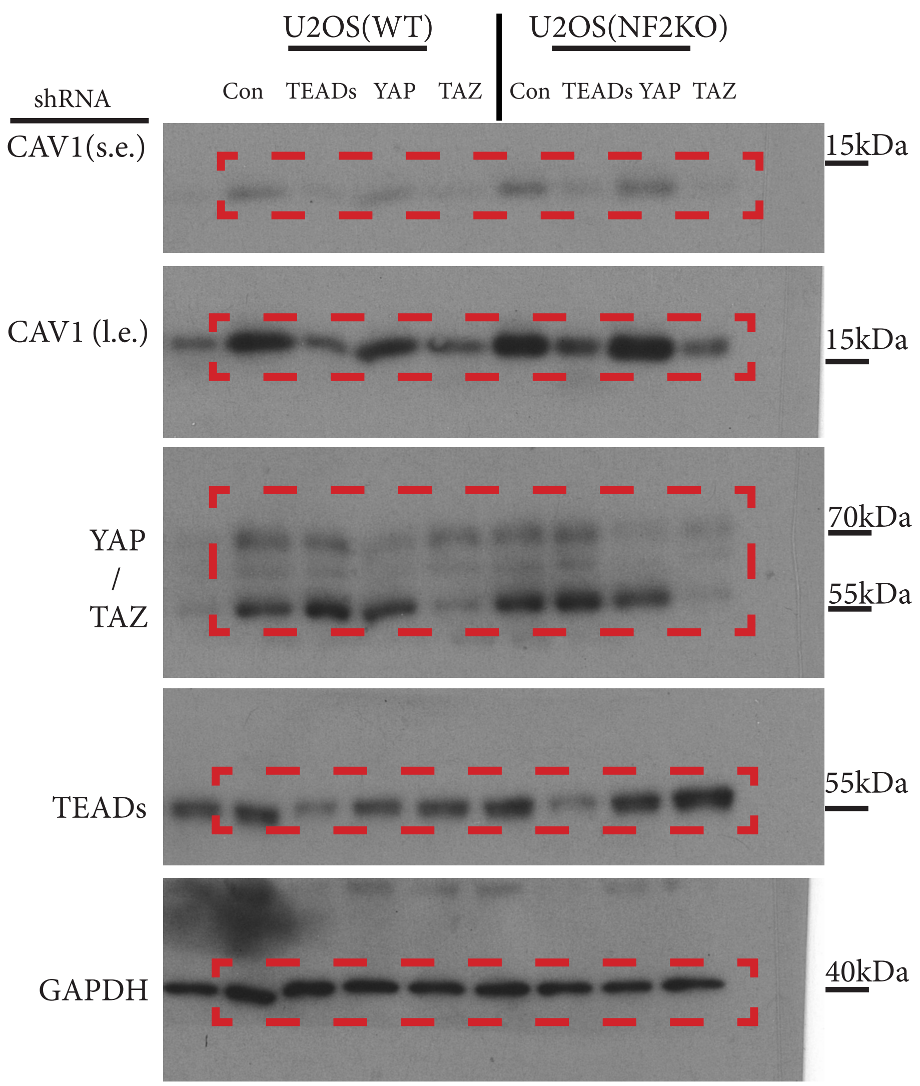

Full scans of WBs shown in Figure S5D

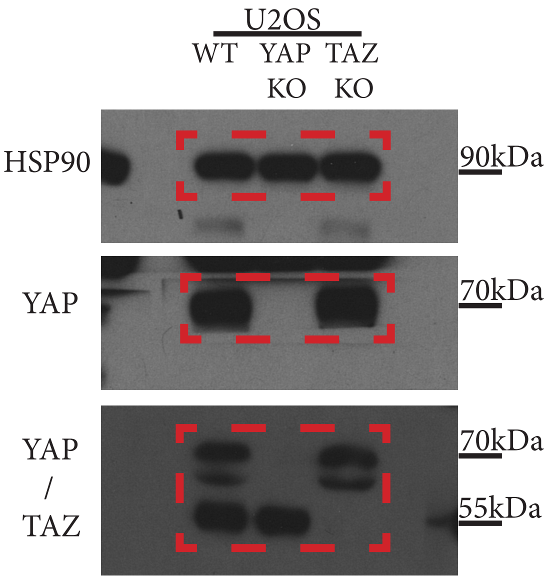

Full scans of WBs shown in Figure S5E

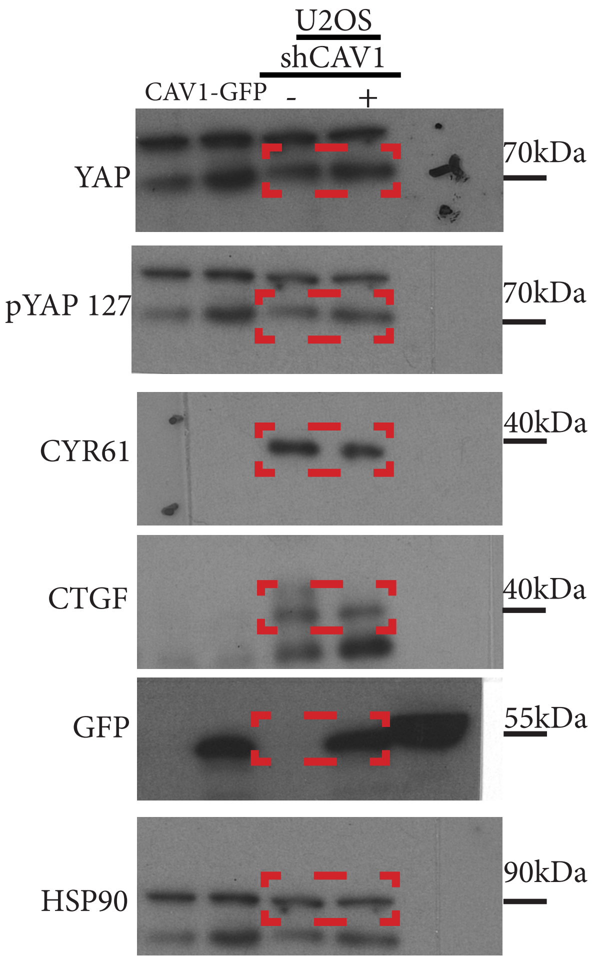

Full scans of WBs shown in Figure S5F

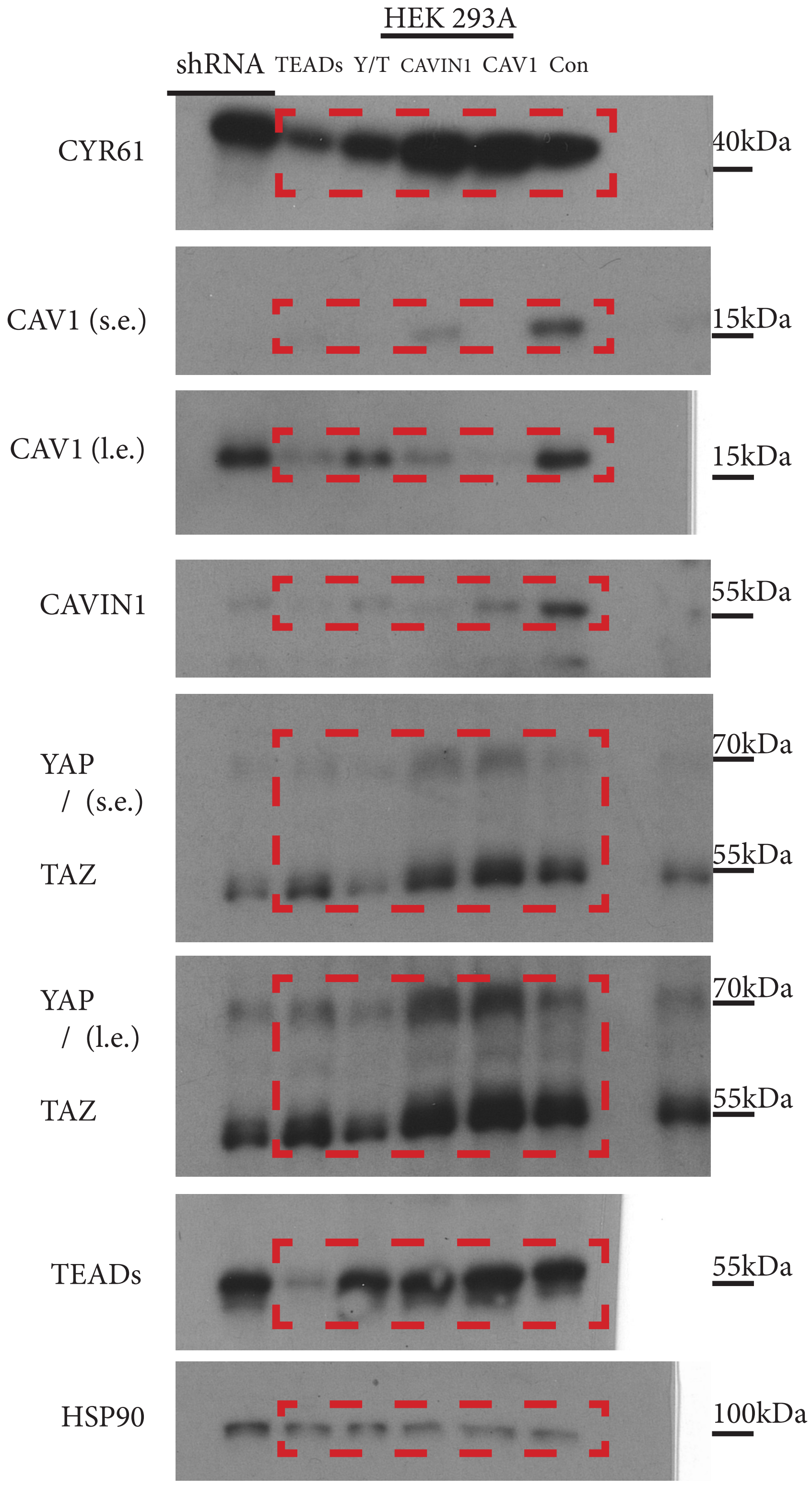

Full scans of WBs shown in Figure S6G

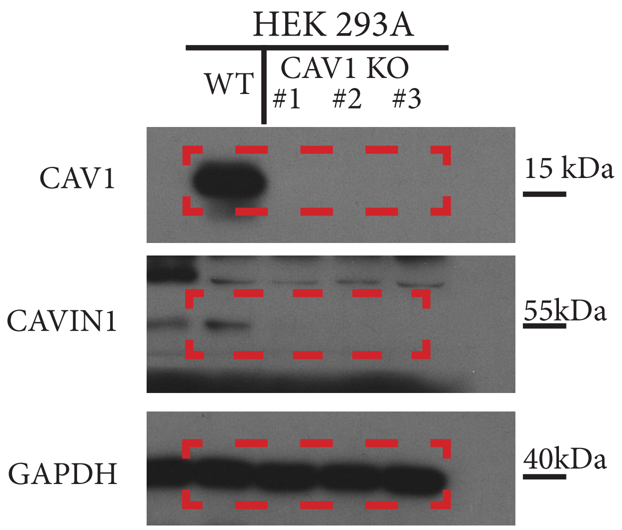

Full scans of WBs shown in Figure S6H

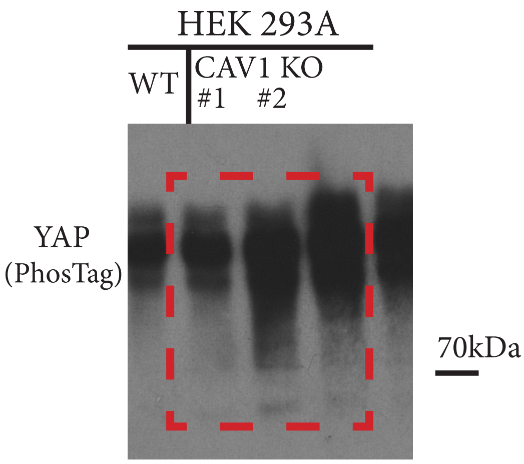

Full scans of WBs shown in Figure S6I

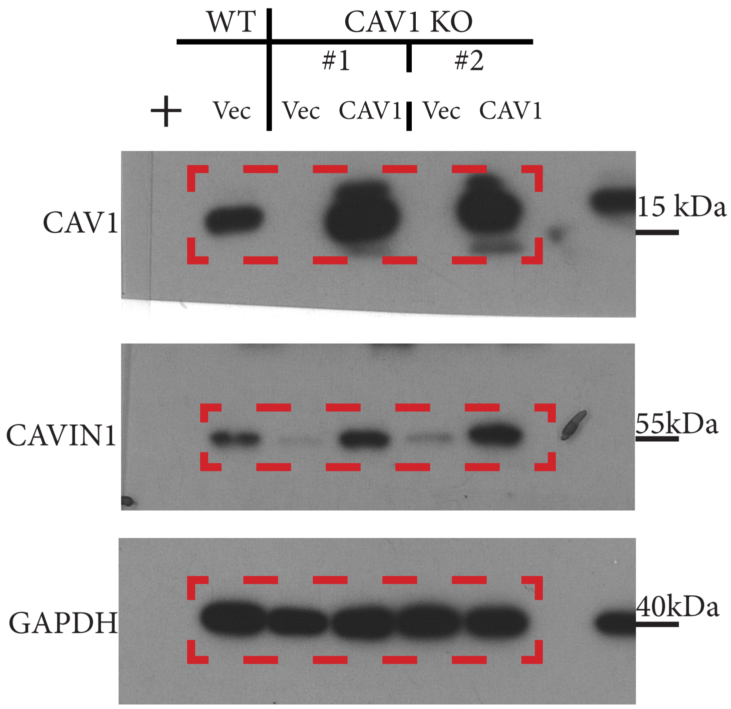

Full scans of WBs shown in Figure S7N

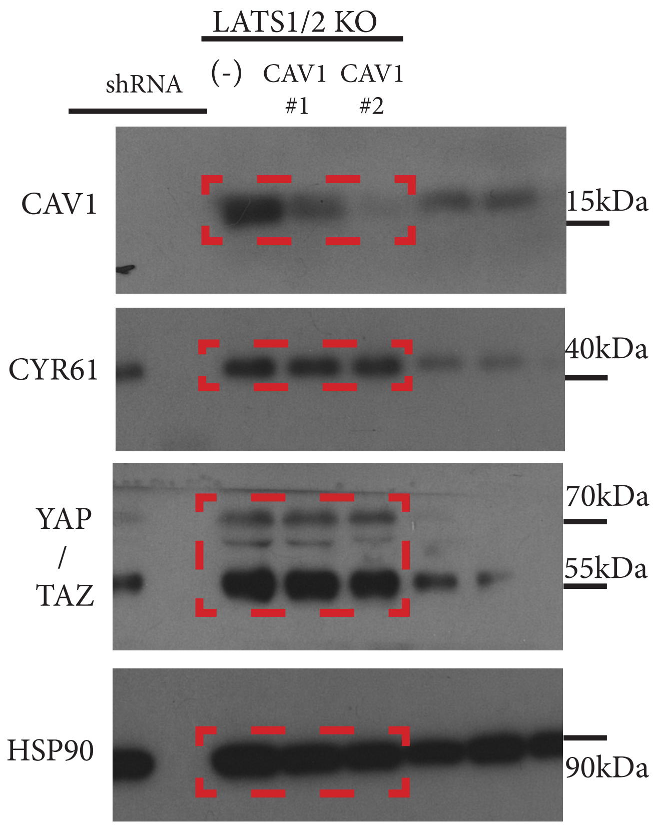

Supplement: Data S1. Full Scans of WBs Shown in the Figures [file mmc2.pdf]
